# Matching-adjusted indirect treatment comparison of liso-cel versus axi-cel in relapsed or refractory large B cell lymphoma

**DOI:** 10.1186/s13045-021-01144-9

**Published:** 2021-09-08

**Authors:** David G. Maloney, John Kuruvilla, Fei Fei Liu, Ana Kostic, Yeonhee Kim, Ashley Bonner, Yixie Zhang, Christopher P. Fox, Guillaume Cartron

**Affiliations:** 1 Fred Hutchinson Cancer Research Center, 1100 Fairview Ave N, Seattle, WA 98109-1024 USA; 2 Princess Margaret Cancer Centre, Toronto, ON Canada; 3 Bristol-Myers Squibb, Princeton, NJ USA; 4 Bristol-Myers Squibb, Seattle, WA USA; 5 EVERSANA, Burlington, ON Canada; 6 Nottingham University Hospitals NHS Trust, Nottingham, UK; 7 Montpellier University Hospital Center, Montpellier, France

**Keywords:** CAR T cell therapy, Lisocabtagene maraleucel, Axicabtagene ciloleucel, Indirect treatment comparison, Matching-adjusted indirect comparison

## Abstract

**Background:**

In the absence of randomized studies directly comparing chimeric antigen receptor T cell therapies, this study used matching-adjusted indirect comparisons (MAIC) to evaluate the comparative efficacy and safety of lisocabtagene maraleucel (liso-cel) versus axicabtagene ciloleucel (axi-cel) in patients with relapsed or refractory large B cell lymphoma (LBCL).

**Methods:**

Primary data sources included individual patient data from the TRANSCEND NHL 001 study (TRANSCEND [NCT02631044]; *N* = 256 for efficacy set, *N* = 269 for safety set) for liso-cel and summary-level data from the ZUMA-1 study (NCT02348216; *N* = 101 for efficacy set, *N* = 108 for safety set) for axi-cel. Inter-study differences in design, eligibility criteria, baseline characteristics, and outcomes were assessed and aligned to the extent feasible. Clinically relevant prognostic factors were adjusted in a stepwise fashion by ranked order. Since bridging therapy was allowed in TRANSCEND but not ZUMA-1, the initial efficacy and safety analyses included bridging therapy use as a matching factor (TRANSCEND patients who received bridging therapy were removed). Subsequent sensitivity analyses excluded this matching factor.

**Results:**

The initial analysis showed similar MAIC-weighted efficacy outcomes between TRANSCEND and ZUMA-1 for overall and complete response rates (odds ratio [95% confidence interval (CI)], 1.40 [0.56–3.49] and 1.21 [0.56–2.64], respectively) and for overall survival and progression-free survival (hazard ratio [95% CI], 0.81 [0.44–1.49] and 0.95 [0.58–1.57], respectively). MAIC-weighted safety outcomes favored liso-cel, with significantly lower odds of all-grade and grade ≥ 3 cytokine release syndrome (odds ratio [95% CI], 0.03 [0.01–0.07] and 0.08 [0.01–0.67], respectively) and study-specific neurological events (0.16 [0.08–0.33] and 0.05 [0.02–0.15], respectively). Efficacy and safety outcomes remained similar in sensitivity analyses, which did not include use of bridging therapy as a matching factor.

**Conclusions:**

After matching and adjusting for clinically relevant prognostic factors, liso-cel demonstrated comparable efficacy and a more favorable safety profile compared with axi-cel in patients with third- or later-line relapsed or refractory LBCL.

Trial registration: NCT02631044 and NCT02348216

**Supplementary Information:**

The online version contains supplementary material available at 10.1186/s13045-021-01144-9.

## Background

Non-Hodgkin lymphoma (NHL) is a common form of cancer, accounting for 2.8% of new cases and 2.6% of deaths in 2018 from cancer worldwide [1]. Diffuse large B cell lymphoma (DLBCL) is the most common NHL subtype and is included in a group of aggressive lymphomas referred to as large B cell lymphomas (LBCL). DLBCL accounts for approximately 30% of all lymphoid malignancies and 37% of all B cell lymphomas worldwide [2, 3]. Many patients achieve durable remission with chemoimmunotherapy, yet up to 50% eventually have relapsed or refractory (R/R) disease [4]. Treatment options are limited for patients with R/R LBCL and treatment outcomes in the third- or later-line setting are historically poor [5, 6].

Chimeric antigen receptor (CAR) T cell therapies have shown clinical activity in patients with R/R LBCL, with objective response rates (ORR) and complete response rates (CRR) ranging from 52 to 82% and from 40 to 54%, respectively [7–9]. The following 3 CAR T cell therapies are available for third- or later-line treatment of LBCL: tisagenlecleucel, axicabtagene ciloleucel (axi-cel), and lisocabtagene maraleucel (liso-cel) [10–14]. All 3 products are CD19-directed CAR T cell therapies; liso-cel and tisagenlecleucel have a 4-1BB costimulatory domain, whereas axi-cel has a CD28 costimulatory domain [7, 8, 15]. Liso-cel is a defined composition CAR T cell product administered at equal target doses of CD8^+^ and CD4^+^ CAR^+^ T cells at the planned dose [7].

As no head-to-head studies comparing CD19-directed CAR T cell therapies have been conducted to inform treatment decisions, methods for indirect treatment comparisons can be used to guide comparison between individual studies. We conducted 2 separate indirect treatment comparisons for liso-cel versus axi-cel and tisagenlecleucel; the comparison with tisagenlecleucel will be reported separately. The analysis reported here used matching-adjusted indirect comparisons (MAIC) to estimate the comparative efficacy and safety of liso-cel from the TRANSCEND NHL 001 study (TRANSCEND) relative to axi-cel using data from the ZUMA-1 study for third- or later-line treatment of R/R LBCL.

## Methods

### Data sources

The primary data source for liso-cel was individual patient data (IPD), which the authors had access to, from the TRANSCEND study (data cutoff: August 12, 2019). TRANSCEND (NCT02631044) is a phase 1, single-arm, multicenter, seamless design study evaluating the efficacy and safety of liso-cel as third- or later-line treatment for patients with R/R LBCL [7]. Patients with LBCL, which included DLBCL not otherwise specified (de novo or transformed from follicular lymphoma [FL] or other indolent lymphomas), high-grade B cell lymphoma with *MYC* and *BCL2* and/or *BCL6* rearrangements with DLBCL histology, primary mediastinal B cell lymphoma, and FL grade 3B, were eligible if they had R/R positron emission tomography (PET)–positive disease after ≥ 2 lines of prior therapy, including treatment with an anti-CD20–targeted agent and anthracycline. Other inclusion criteria included an Eastern Cooperative Oncology Group performance status (ECOG PS) of 0–2, and adequate organ function. Patients with secondary central nervous system (CNS) involvement and prior autologous or allogeneic hematopoietic stem cell transplantation (auto-HSCT; allo-ASCT) were permitted. Patients with primary CNS lymphoma were excluded. Primary endpoints were adverse events (AE) and ORR as assessed by an independent review committee (IRC). Secondary endpoints included CRR, duration of response, progression-free survival (PFS) per IRC assessment, and overall survival (OS).

A literature review identified the ZUMA-1 study as the key source of efficacy and safety data for axi-cel as a treatment option for R/R LBCL. The primary source of data for axi-cel was a peer-reviewed publication reporting summary-level data from the ZUMA-1 study (data cutoff: August 11, 2018) [16]. Secondary data sources for ZUMA-1, which included the YESCARTA European Medicines Agency public assessment report, summary of product characteristics, and Biologics License Application clinical review memorandum [17–19], were consulted only when data were unavailable in the primary publication or when additional clarity was required. ZUMA-1 (NCT02348216) was a phase 1/2, single-arm, multicenter, registrational study evaluating the efficacy and safety of axi-cel in patients with R/R LBCL [16]. Patients with histologically confirmed DLBCL, primary mediastinal B cell lymphoma, or DLBCL transformed from FL were eligible if they had R/R disease after 2 systemic lines of therapy, chemotherapy-refractory disease (no response to last line of therapy and/or refractory after auto-HSCT), had previously received an anti-CD20 monoclonal antibody-containing regimen and an anthracycline-containing chemotherapy, and had an ECOG PS of 0–1. Patients were excluded if they had secondary CNS lymphoma or prior allo-HSCT. The primary endpoints were safety for phase 1 and ORR per investigator’s assessment for phase 2. Key secondary endpoints were ORR per IRC, PFS, OS, and duration of response. Among 108 patients who received axi-cel, 101 were evaluable in the phase 2 study.

The TRANSCEND LBCL efficacy set (*N* = 256), defined as liso-cel-treated patients who had confirmed PET-positive disease before liso-cel administration, was used for liso-cel efficacy outcomes (ORR, CRR, PFS, and OS) [7]. The ZUMA-1 phase 2 modified intention-to-treat set (*N* = 101), defined as patients enrolled in phase 2 (ie, had PET-positive disease at baseline) who received axi-cel, was used for axi-cel efficacy outcomes [8, 16]. For safety outcomes, the TRANSCEND LBCL-treated set (*N* = 269), defined as all patients with LBCL who received liso-cel, was used for liso-cel and the ZUMA-1 phase 1/2 safety analysis set, defined as all patients who received axi-cel in both phases 1 and 2, was used for axi-cel (*N* = 108) [7, 16].

### Study comparisons

Comparison of TRANSCEND versus ZUMA-1 study designs, eligibility criteria, and baseline characteristics showed sufficient inter-study similarities to allow for comparison (Table 1). However, imbalances in patient populations enrolled between studies necessitated a MAIC analysis to reduce bias when indirectly comparing liso-cel to axi-cel.

**Table 1:** Study design characteristics and inclusion criteria for TRANSCEND versus ZUMA-1

Key study design features	TRANSCEND (liso-cel)	ZUMA-1 (axi-cel)
Phase	1	1/2
Design	Single arm	Single arm
Blinding	Open label	Open label
Centers	Multicenter	Multicenter
Country	United States	Multiple (Israel and United States)
Bridging therapy	Allowed	Not allowed
PET-positive disease after bridging therapy	Confirmed	NA
LDC	Yes	Yes
Regimen and dosage of LDC	FLU (30 mg/m^2^/day for 3 d) and CY (300 mg/m^2^/day for 3 d), completed 2–7 d before infusion	FLU (30 mg/m^2^) and CY (500 mg/m^2^) on the fifth, fourth, and third day before infusion
CAR T cell regimen and dosage	DL1S: 50 × 10^6^ CAR^+^ T cells (25 × 10^6^ CD8^+^ CAR^+^ T cells and 25 × 10^6^ CD4^+^ CAR^+^ T cells) DL1D: 50 × 10^6^ CAR^+^ T cells DL2S: 100 × 10^6^ CAR^+^ T cells (50 × 10^6^ CD8^+^ CAR^+^ T cells and 50 × 10^6^ CD4^+^ CAR^+^ T cells) DL3S: 150 × 10^6^ CAR^+^ T cells (75 × 10^6^ CD8^+^ CAR^+^ T cells and 75 × 10^6^ CD4^+^ CAR^+^ T cells)	Single infused dose of 2 × 10^6^ CAR T cells per kg of body weight, with a maximum permitted dose of 2 × 10^8^ CAR T cells

Key inclusion criteria	TRANSCEND (liso-cel)	ZUMA-1 (axi-cel)	Action taken in TRANSCEND IPD and rationale
NHL subtypes	DLBCL NOS, HGBCL, tFL, tiNHL, PMBCL, FL3B	DLBCL NOS,* HGBCL, PMBCL, tFL	Recategorized TRANSCEND to align with ZUMA-1 definition for DLBCL to retain TRANSCEND patients. Specifically, DLBCL NOS, HGBCL, and tiNHL from TRANSCEND were grouped together in “DLBCL” for comparison with “DLBCL” category in ZUMA-1
Age, years	≥ 18	≥ 18	None
ECOG PS	≤ 2^†^	≤ 1	None
Prior lines of treatment	≥ 2	≥ 2^‡^	Redefined in TRANSCEND such that salvage chemotherapy and auto-HSCT were considered as 2 separate lines of therapy to align with ZUMA-1 definition
Prior auto-HSCT	Allowed	Allowed, but not within 6 weeks of infusion	None
Prior allo-HSCT	Allowed (not within 90 d of leukapheresis)	Not allowed	None
Prior regimen required	Anthracycline and rituximab (or other CD20-targeted agents)	Anti-CD20 monoclonal antibody unless investigator determines that tumor is CD20 negative, and an anthracycline-containing chemotherapy regimen	None
Response to prior therapy	R/R disease after ≥ 2 lines of prior therapy or after auto-HSCT	No response to first-line therapy (primary refractory disease) OR no response to second- or later-line of therapy OR refractory after auto-HSCT (disease progression or relapsed ≤ 12 mo of auto-HSCT)	
R/R to last therapy	Refractory: best response to last therapy as progressive disease, stable disease, or PR Relapsed: best response to last therapy as CR	Refractory: best response to last therapy^§^ as progressive disease or stable disease Relapsed: best response to last therapy^§^ of PR or CR	Redefined in TRANSCEND to align with ZUMA-1 definition. Specifically, in TRANSCEND, % refractory to last therapy was rederived to include progressive disease and stable disease, whereas % relapse was rederived to include PR and CR
Absolute lymphocyte count	No minimum requirement^¶^	≥ 100/μL	Redefined in TRANSCEND to align with ZUMA-1 definition
Absolute neutrophil count	No minimum requirement^¶^	≥ 1000/μL	None
Platelet count	No minimum requirement^¶^	≥ 75,000/μL	None
Hemoglobin	No minimum requirement^¶^	Not reported	None
Alanine aminotransferase	≤ 5 × ULN	≤ 2.5 × ULN	None
Total bilirubin	< 2.0 mg/dL	≤ 1.5 mg/dL	None
Serum creatinine	≤ 1.5 × ULN	Not reported	None
CrCl	> 30 mL/min/1.73 m^2^ (Cockcroft-Gault)	≥ 60 mL/min (Cockcroft-Gault)	Redefined in TRANSCEND to align with ZUMA-1 definition
Dyspnea	Grade ≤ 1 by NCI CTCAE	Not clinically significant	None
Oxygen saturation	≥ 92% on room air	> 92% on room air	None
LVEF	≥ 40%	≥ 50%	Redefined in TRANSCEND to align with ZUMA-1 definition
Active CNS involvement	Secondary CNS involvement allowed	Not allowed	None
History of another primary malignancy	Not allowed unless another primary malignancy has been in remission for ≥ 2 y	Not allowed unless disease free for ≥ 3 y	None
Infections	Uncontrolled systemic fungal, bacterial, viral, or other infection despite appropriate antibiotics or other treatment at the time of leukapheresis or liso-cel administration	Presence of fungal, bacterial, viral, or other infection that is uncontrolled or requiring IV antimicrobials for management	None
Cardiovascular conditions or clinically significant cardiac disease	Within 6 mo of screening/enrollment	Within 12 mo of enrollment	None

Allo-HSCT, allogeneic hematopoietic stem cell transplantation; auto-HSCT, autologous hematopoietic stem cell transplantation; axi-cel, axicabtagene ciloleucel; CAR, chimeric antigen receptor; CNS, central nervous system; CrCl, creatinine clearance; CY, cyclophosphamide; DLBCL, diffuse large B cell lymphoma; DL1S, dose level 1 (single dose); DL1D, dose level 1 (double dose); DL2S, dose level 2 (single dose); DL3S, dose level 3 (single dose); ECOG PS, Eastern Cooperative Oncology Group performance status; FL3B, follicular lymphoma grade 3B; FLU, fludarabine; HGBCL, high-grade B cell lymphoma; liso-cel; lisocabtagene ciloleucel; LDC, lymphodepleting chemotherapy; LVEF, left ventricular ejection fraction; NA, not applicable; NCI CTCAE, National Cancer Institute Common Terminology Criteria for Adverse Events; NHL, non-Hodgkin lymphoma; NOS, not otherwise specified; PET, positron emission tomography; PMBCL, primary mediastinal B cell lymphoma; R/R, relapsed or refractory; tFL, transformed follicular lymphoma; tiNHL, transformed indolent non-Hodgkin lymphoma; ULN, upper limit of normal

^*^ZUMA-1 histology was classified according to WHO 2008 classification; tiNHL was included under DLBCL NOS histology per WHO 2008 [39] and patients with tiNHL were included in ZUMA-1 per study protocol [16]

^†^ECOG PS of 2 was allowed until Protocol Amendment 5, August 17, 2017 [7]

^‡^Per ZUMA-1 ClinicalTrials.gov record (NCT02348216)

^§^ZUMA-1 did not report a definition of “last therapy,” thus, was assumed as any therapy received by the patient before entering study

^¶^Assessed by the investigator to have had adequate bone marrow function to receive LDC

#### Study designs

Both studies were multicenter, single-arm studies, and both used similar lymphodepleting chemotherapy (fludarabine and cyclophosphamide; Table 1), although the doses were higher in the ZUMA-1 study. The studies differed in their allowance of bridging therapy use (ie, not allowed in ZUMA-1) and CAR T cell dosage (defined composition and fixed dose in TRANSCEND, weight-based dose in ZUMA-1). In addition, the liso-cel dose is based on post-thaw measures of viable cell count and CAR^+^ T cell frequency that is used to calculate the corresponding required dose volume to be infused at the administration site, whereas the full weight-based dose of axi-cel is administered, regardless of post-thaw recovery. Eligibility criteria differed between trials for prior allo-HSCT, secondary CNS involvement, degree of impaired renal and cardiac function, and minimum hematologic parameter requirements (Table 1). Notably, the studies also differed in their enrollment processes; ZUMA-1 did not permit enrollment and leukapheresis unless a CAR T cell manufacturing slot was available, whereas TRANSCEND allowed bridging therapy after leukapheresis at the discretion of the treating clinician during the liso-cel manufacturing process [7, 20]. The median (range) time from leukapheresis to product availability was 24 (17–51) days in TRANSCEND and 17 (14–51) days in ZUMA-1 (7, 17).

#### Patient characteristics

Of 18 baseline patient characteristics reported in both studies, definitions (tumor burden, disease histology, number of lines of prior therapy, and R/R to last therapy), categorization (International Prognostic Index score), and/or minimum/maximum thresholds (creatinine clearance [CrCl] before lymphodepleting chemotherapy, left ventricular ejection fraction [LVEF] at screening, and absolute lymphocyte count before leukapheresis) differed between the studies for 8 patient characteristics (Table 2). For safety outcomes, 11 baseline patient characteristics were considered, including the following 3 unique factors for safety: baseline grade ≥ 3 anemia, neutropenia, and thrombocytopenia. Differences between studies were aligned by reclassifying or recalculating the corresponding variables within the TRANSCEND IPD to match classifications or definitions reported in ZUMA-1. Definitions and/or categorizations for the remaining patient characteristics were similar and, therefore, did not require alignment.

**Table 2:** Comparison of clinical factors and SMDs before and after MAIC for OS

Clinical factor	OS						
	ZUMA-1 (axi-cel) phase 2 mITT set	TRANSCEND (liso-cel) LBCL efficacy set					
		Before MAIC (naïve)		After MAIC (SA1)		After MAIC (SA2)	
N/ESS	*N* = 101	*N* = 256		ESS = 152.6		ESS = 98.9	
	Stat	Stat	SMD	Stat	SMD	Stat	SMD
Age, years, mean (SD)	56.3 (12.0)	60.3 (13.3)	0.308	56.3 (12.0)	0.000	56.3 (12.0)	0.000
Male sex, %	67.3	66.0	0.027	68.3	0.020	67.3	0.000
IPI score, %*							
0–2	54.5	58.6	0.162	54.5	0.000	54.5	0.000
3–4	45.5	39.8		45.5		45.5	
5	0.0	0.8		0.0		0.0	
Missing	0.0	0.8		0.0		0.0	
ECOG PS at screening, %							
0	41.6	40.6	0.178	37.9	0.075	41.6	0.000
1	58.4	57.8		62.1		58.4	
2	0.0	1.6		0.0		0.0	
Disease stage, %							
I or II	14.9	27.0	0.304	23.5	0.219	14.9	0.000
III or IV	85.1	72.3		76.5		85.1	
Missing	0.0	0.8		0.0		0.0	
Tumor burden based on SPD before LDC, cm^2^, mean (SD)^†^	50.4 (43.7)	43.7 (48.1)	0.142	50.4 (43.8)	0.000	50.4 (43.9)	0.000
Secondary CNS disease at time of treatment, %							
No	100.0	97.7	0.219	100.0	0.000	100.0	0.000
Yes	0.0	2.3		0.0		0.0	
Extranodal disease, %							
No	30.7	46.9	0.344	42.9	0.255	30.7	0.000
Yes	69.3	52.3		57.1		69.3	
Missing	0.0	0.8		0.0		0.0	
Bulky disease, %							
No	83.2	87.9	0.155	83.2	0.000	83.2	0.000
Yes	16.8	11.3		16.8		16.8	
Missing	0.0	0.8		0.0		0.0	
Disease histology, %							
DLBCL^‡^	76.2	71.1	0.242	76.3	0.000	76.3	0.000
DLBCL tFL	15.8	22.3		15.8		15.8	
PMBCL	7.9	5.5		7.9		7.9	
FL3B	0.0	1.2		0.0		0.0	
No. of lines of prior therapy, %^§^							
1	3.0	0.8	0.179	0.2	0.228	3.0	0.000
2	27.7	25.0		25.9		27.7	
≥ 3	69.3	73.8		73.9		69.3	
Missing	0.0	0.4		0.0		0.0	
Prior allo-HSCT, %	0.0	2.7	0.237	0.0	0.000	0.0	0.000
Prior auto-HSCT, %	24.8	33.2	0.186	31.4	0.148	24.8	0.000
Bridging therapy, %							
No	100.0	41.4	1.682	35.0	1.925	35.9	1.889
Yes	0.0	58.6		65.0		64.1	
R/R to last therapy, %^¶^							
Relapsed	20.8	35.9	0.359	20.8	0.000	20.8	0.000
Refractory	79.2	61.7		79.2		79.2	
Missing	0.0	2.3		0.0		0.0	
CrCl before LDC, %^#^							
< 60 mL/min	0.0	19.1	0.688	13.4	0.557	0.0	0.000
≥ 60 mL/min	100.0	80.9		86.8		100.0	
LVEF at screening, %^#^							
< 50%	0.0	5.1	0.327	4.3	0.300	0.0	0.000
≥ 50%	100.0	94.9		95.7		100.0	
Pre-leukapheresis ALC (10^9^/L), %^#^							
< 0.1	0.0	0.4	0.091	0.2	0.067	0.0	0.000
≥ 0.1	100.0	94.1		94.2		100.0	
Missing	0.0	5.5		5.6		0.0	
Statistics, %							
Factors with SMD < 0.2	NA	44.4	NA	66.7	NA	94.4	NA
Factors with SMD < 0.1	NA	11.1	NA	61.1	NA	94.4	NA

ALC, absolute lymphocyte count; allo-HSCT, allogeneic hematopoietic stem cell transplantation; auto-HSCT, autologous hematopoietic stem cell transplantation; axi-cel, axicabtagene ciloleucel; CNS, central nervous system; CrCl, creatinine clearance; DLBCL, diffuse large B cell lymphoma; ECOG PS, Eastern Cooperative Oncology Group performance status; ESS, effective sample size; FL3B, follicular lymphoma grade 3B; HGBCL, high-grade B cell lymphoma; IPI, International Prognostic Index; LBCL, large B cell lymphoma; liso-cel, lisocabtagene maraleucel; LDC, lymphodepleting chemotherapy; LVEF, left ventricular ejection fraction; MAIC, matching-adjusted indirect comparison; mITT, modified intention to treat; N, sample size; NA, not applicable; NOS, not otherwise specified; OS, overall survival; PMBCL, primary mediastinal B cell lymphoma; R/R, relapsed or refractory; SA1, sensitivity analysis 1; SA2, sensitivity analysis 2; SD, standard deviation; SMD, standard mean difference; SPD, sum of the product of perpendicular diameters; Stat, statistic; tFL, transformed follicular lymphoma; tiNHL, transformed indolent non-Hodgkin lymphoma

^*^Per ZUMA-1 categorization

^†^Per investigator assessment

^‡^Includes DLBCL NOS, HGBCL, and tiNHL for TRANSCEND; includes DLBCL NOS and HGBCL for ZUMA-1

^§^Per ZUMA-1, salvage chemotherapy and auto-HSCT were considered separate regimens

^¶^Per ZUMA-1, refractory was defined as best response to last therapy of progressive disease or stable disease and relapsed defined as best response to last therapy of partial response or complete response

^#^Per ZUMA-1 eligibility criteria, all patients enrolled in ZUMA-1 had CrCl ≥ 60 mL/min before LDC, LVEF ≥ 50% at screening, and pre-leukapheresis ALC ≥ 0.1 × 10^9^/L

#### Definitions of outcome measures

Four efficacy outcomes and 10 selected safety outcomes were assessed in this analysis. The efficacy outcomes evaluated included ORR, CRR, PFS, and OS. For ORR and CRR assessments, although the primary endpoint for ZUMA-1 was investigator-assessed ORR, both studies reported response outcomes by IRC based on PET/computed tomography scans, which were used for comparisons; however, ZUMA-1 used the revised International Working Group criteria [21], whereas TRANSCEND used the more recent Lugano classification [22]. As PET-based assessment was used in both studies and any uncertain responses would be subject to additional testing, inter-study differences in ORR and CRR assessment criteria were anticipated to be minimal. Definitions for PFS and OS were similar. For PFS, both studies captured investigator- and IRC-assessed events and utilized the same censoring rules.

The safety outcomes evaluated CAR T cell AEs of interest, including all-grade and grade ≥ 3 cytokine release syndrome (CRS) by Lee et al. [23] criteria and neurological events (NE), including aphasia and encephalopathy; grade ≥ 3 infections; all-grade hypogammaglobulinemia; and grade ≥ 3 prolonged cytopenia, including anemia, neutropenia, and thrombocytopenia. Both studies had a similar treatment-emergent AE reporting window (90 days after infusion in TRANSCEND and 92 days after infusion in ZUMA-1) and had similar definitions for CRS, infections, and hypogammaglobulinemia, whereas definitions for study-specific NEs, including grouped terms of encephalopathy and aphasia, and prolonged cytopenias varied between studies. Despite potential differences in study-specific NE definitions, NEs reported by each study were investigator-identified neurological AEs considered clinically relevant and related to CAR T cell therapy. In both studies, infections were defined using high-level group terms per the Infections and Infestations system organ class and hypogammaglobulinemia was defined using grouped preferred terms assessed as AEs by investigators. Prolonged cytopenia was defined as grade ≥ 3 anemia, neutropenia, or thrombocytopenia not resolved by Day 29 (TRANSCEND) or by Day 30 (ZUMA-1); however, reporting was based on laboratory values in TRANSCEND and on AEs reported by the investigator in ZUMA-1. Therefore, MAIC was not conducted for prolonged cytopenia and an unadjusted, side-by-side descriptive comparison was done to describe reported data. However, grade ≥ 3 prolonged anemia, neutropenia, and thrombocytopenia were individually reported as AEs by investigators in both studies and were therefore compared via MAIC.

No outcomes were rederived from TRANSCEND for alignment with ZUMA-1.

### Statistical analysis

As no common comparator was available, unanchored MAICs were conducted to determine the relative efficacy and safety of liso-cel (TRANSCEND) versus axi-cel (ZUMA-1) [24]. Generalized linear models were used to estimate odds ratios (OR) for binary outcomes (ie, ORR, CRR, and safety outcomes), and Cox proportional hazards models were used to estimate hazard ratios (HR) for time-to-event outcomes (ie, OS and PFS).

Relevant clinical factors for matching and adjusting were identified and ranked via a literature search followed by input from a panel of 5 external clinical experts (Canada, France, Germany, UK, and US) with CAR T cell therapy experience. A final ranked list of clinical factors was derived per outcome by evaluating the strength of association of each clinical factor to each outcome endpoint using statistical random forest models (efficacy endpoints only) and incorporating clinical expert rankings to create an evidence-informed clinical ranking [25–27]. A single list of ranked factors was used for all safety outcomes, based on a literature search and consensus among clinical experts.

For each comparison, patients from TRANSCEND were removed from the IPD set if they did not satisfy the eligibility criteria and treatment protocol of ZUMA-1. Specifically, patients with prior allo-HSCT, ECOG PS of 2, secondary CNS involvement, or bridging therapy use were removed from the TRANSCEND IPD set as these patients were ineligible for ZUMA-1. In addition, as FL grade 3B was not expected to differ from the other histologies with respect to safety effects, patients with FL grade 3B were removed from the TRANSCEND IPD set for efficacy outcomes only. As patients in ZUMA-1 were ineligible to receive bridging therapy, initial analyses of liso-cel versus axi-cel involved removing patients from the TRANSCEND IPD set who had received bridging therapy (ie, matching on bridging therapy use). As bridging therapy use could be potentially associated with more aggressive disease or with differences in product manufacturing time between trials, and given the lack of consensus among clinicians regarding bridging therapy use as a matching factor in these analyses, sensitivity analyses were also conducted without matching on bridging therapy use (ie, TRANSCEND patients who received bridging therapy were maintained in the analysis).

After completing the MAIC matching phase, the remaining patients from TRANSCEND were weighted using a method-of-moments propensity score algorithm, which was chosen because only summary-level data were available from ZUMA-1 and this method guarantees an exact balancing of clinical factors between comparison studies of interest. For binary endpoints, estimates of the comparative efficacy of liso-cel versus axi-cel were derived from an intercept-only logistic regression with MAIC adjustment weights. The intercept represents a prediction of the log odds of the outcome of interest if a typical patient from ZUMA-1 had received liso-cel. An estimate of the OR for liso-cel versus axi-cel was derived as the ratio between the estimated odds for liso-cel from weighted IPD and the estimated odds from summary-level data for ZUMA-1. Therefore, the relative probabilities of response or AEs between liso-cel and axi-cel are expressed and presented as ORs. For time-to-event endpoints, comparative efficacy of liso-cel versus axi-cel was estimated as HRs derived from a weighted Cox proportional hazards model with a binary treatment indicator (ie, liso-cel vs axi-cel). To fit this model under usual circumstances, IPD from both trials would be required. In place of IPD for ZUMA-1, pseudo-IPD for PFS and OS were derived by digitizing published Kaplan–Meier survival curves and using the Guyot et al. 2012 [28] approach. In turn, medians and 95% confidence intervals (CI) for ZUMA-1 were estimated from this pseudo-IPD. In the Cox regression model, TRANSCEND IPD values were assigned propensity score weights as defined above, while pseudo-IPD values for ZUMA-1 were left unweighted (weights for pseudo-observations were set to 1).

For a given set of ranked clinical factors, separate MAICs were conducted sequentially, adjusting for 1 additional variable at a time in order of ranked importance. After fitting each model, the performance and suitability of each MAIC model was assessed based on the following criteria: effective sample size (ESS; a proxy for sample size when patients are weighted), distribution of patient weights, summary statistics (assessment of balance between study populations), and assumption of proportional hazards for OS and PFS. Balance was assessed using the absolute value of the standardized mean difference (SMD) for each factor. An SMD ≥ 0.10 was considered indicative of potentially important imbalances between comparisons [29]. For a given factor, a reduction in the SMD after matching and adjusting signified a reduction in imbalance between studies.

Initial analyses of efficacy outcomes were conducted for patients who did not receive bridging therapy (ie, matched for bridging therapy use). Overall, 10 clinical factors were included in this analysis. First, the 5 clinical factors that related to study eligibility and treatment protocol were used as matching criteria (bridging therapy use, disease histology, ECOG PS, secondary CNS involvement, and prior allo-HSCT). An additional 5 clinical factors, which varied across outcomes based on evidence-informed clinical rankings (ORR: tumor burden, R/R status to last therapy, prior auto-HSCT, disease stage, and CrCl; CRR: tumor burden, R/R status to last therapy, bulky disease, prior auto-HSCT, and extranodal disease; PFS: tumor burden, International Prognostic Index [IPI] score, R/R status to last therapy, bulky disease, and CrCl; OS: tumor burden, IPI score, R/R status to last therapy, bulky disease, and age), were then adjusted to reduce residual imbalances between studies among matched patients (Additional file 1: Table S1). Two sensitivity analyses were also performed. Sensitivity analysis 1 (SA1) was the same as the initial analysis except that bridging therapy use was removed as a matching factor (ie, included patients from TRANSCEND who received bridging therapy). This analysis was conducted to help assess the effect of bridging therapy on results, recognizing that bridging therapy use could be related to other factors associated with aggressive disease or the time required to manufacture the cellular product. Sensitivity analysis 2 (SA2) removed bridging therapy use as a matching factor and adjusted for additional factors (number of prior therapies, sex, absolute lymphocyte count, and LVEF). This analysis aimed to assess the effect of balancing additional factors while retaining a reasonable ESS upon excluding the bridging therapy factor.

Initial analyses of safety outcomes were conducted for patients who did not receive bridging therapy. Overall, 9 clinical factors were included: 4 factors related to study eligibility criteria and treatment protocol as matching criteria (secondary CNS involvement, bridging therapy use, ECOG PS, and prior allo-HSCT) and 5 additional factors that were adjusted to minimize differences between patients (baseline grade ≥ 3 anemia, neutropenia, and thrombocytopenia; pre-lymphodepleting chemotherapy tumor burden; and number of prior lines of therapy). A sensitivity analysis was performed, which was the same as the initial analysis except that bridging therapy use was removed from the list of matching factors (ie, included patients from TRANSCEND who received bridging therapy).

All analyses were conducted using R (R Core Team, Vienna, Austria), based on the code outlined in the National Institute for Health and Care Excellence Evidence Synthesis Technical Support Document Series [24].

## Results

### Clinical factors before and after matching and adjusting

For efficacy and safety outcomes, clinical factor comparison before matching demonstrated that some factors were similar between TRANSCEND and ZUMA-1 studies (Table 2; Additional file 1: Tables S2–S5). For efficacy outcomes, notable differences (SMDs ≥ 0.1) were observed for age, IPI score, ECOG PS, disease stage, tumor burden, secondary CNS lymphoma, extranodal disease, bulky disease, disease histology, number of lines of prior therapy, prior allo-HSCT and auto-HSCT, bridging therapy use, R/R to last therapy, CrCl < 60 mL/min, and LVEF < 50%. In initial and sensitivity analyses, matching and adjusting patients from TRANSCEND to ZUMA-1 produced substantial improvements in the balance of clinical factors between studies, with SA2, which included the greatest number of factors, showing the largest improvements in balance (94% of 18 efficacy factors with SMDs < 0.1). As both sensitivity analyses included patients who received bridging therapy, which was not permitted in ZUMA-1, substantial imbalances in this factor remained after matching and adjusting on other factors. Similarly, for safety outcomes, notable differences were observed for age, ECOG PS, secondary CNS lymphoma, number of lines of prior therapy, prior allo-HSCT and auto-HSCT, bridging therapy use, and baseline grade ≥ 3 anemia, neutropenia, and thrombocytopenia. After conducting the MAIC, 82% of 11 safety factors had SMDs < 0.1, indicating imbalance between studies was minimized and that the majority of factors important to safety outcomes were well balanced after matching and adjusting.

### Efficacy outcomes

#### Response rates

Overall, the MAIC results showed no statistically significant difference in the probability of response (ORR, CRR) between liso-cel and axi-cel (Table 3). Before conducting MAICs, unadjusted ORRs were similar for liso-cel (72.7% [*N* = 256]) versus axi-cel (74.3% [*N* = 101]; OR 0.92; 95% confidence interval [CI] 0.54–1.55; *P* = 0.753). In the initial analysis that matched and adjusted for 10 factors, including bridging therapy use (ie, TRANSCEND patients who received bridging therapy were removed from the data set), the ORR for liso-cel increased to 80.1% (ESS = 42.1) and the probability of overall response between liso-cel and axi-cel remained similar (OR 1.40; 95% CI 0.56–3.49; *P* = 0.476). In both SA1 (ESS = 150.3), which adjusted for the same factors except bridging therapy use, and SA2 (ESS = 98.9), which adjusted for 17 factors, the ORR for liso-cel was 71.2% and 70.9%, respectively, and the probability of response remained similar between treatments.

**Table 3:** Summary of ORR and CRR comparisons between the TRANSCEND and ZUMA-1 studies

	ZUMA-1 (axi-cel)		TRANSCEND (liso-cel)		Liso-cel versus axi-cel	
	*N* (%)	Response rate, %	*N*/ESS	Response rate, %	OR (95% CI)	*P*
ORR						
Naïve comparison	101	74.3	256	72.7	0.92 (0.54–1.55)	0.753
Initial analysis			42.1	80.1	1.40 (0.56–3.49)	0.476
Sensitivity analysis 1			150.3	71.2	0.85 (0.48–1.52)	0.591
Sensitivity analysis 2			98.9	70.9	0.84 (0.45–1.58)	0.596
CRR						
Naïve comparison	101	54.5	256	53.1	0.95 (0.60–1.50)	0.815
Initial analysis			39.6	59.2	1.21 (0.56–2.64)	0.630
Sensitivity analysis 1			169.1	48.2	0.78 (0.47–1.27)	0.318
Sensitivity analysis 2			98.9	49.5	0.82 (0.47–1.43)	0.483

Axi-cel, axicabtagene ciloleucel; CI, confidence interval; CRR, complete response rate; ESS, effective sample size; liso-cel, lisocabtagene maraleucel; N, sample size; OR, odds ratio; ORR, objective response rate

Likewise, unadjusted CRRs were similar for liso-cel (53.1%) versus axi-cel (54.5%; OR 0.95; 95% CI 0.60–1.50; *P* = 0.815; Table 3). In the initial analysis, the CRR for liso-cel increased to 59.2% (ESS = 39.6) and the probability of complete response between liso-cel and axi-cel remained similar (OR 1.21; 95% CI 0.56–2.64; *P* = 0.630). In SA1 and SA2, the CRR for liso-cel was 48.2% and 49.5%, respectively, and probability of complete response remained similar between treatments.

#### Survival outcomes

**Fig. 1:**
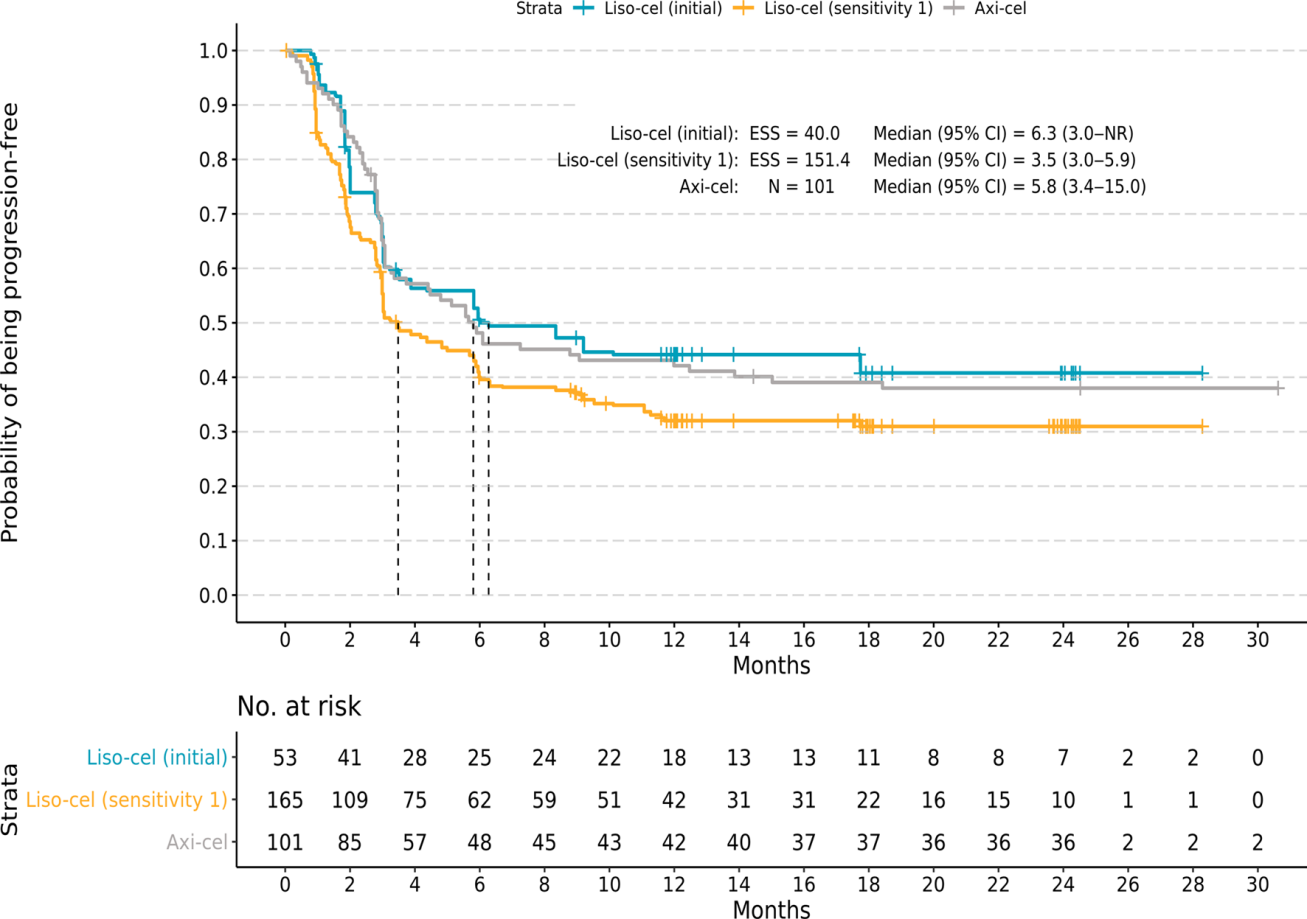
Kaplan–Meier curves for progression-free survival. Kaplan–Meier curves are shown for the initial and sensitivity analysis comparisons with liso-cel versus axi-cel for infused patients. Kaplan–Meier curves for the initial analysis, which matched and adjusted for 10 factors, including bridging therapy use, demonstrated similar cumulative probabilities of progression-free survival for liso-cel and axi-cel. Similar results were observed for sensitivity analysis 1, which was the same as the initial analysis except that bridging therapy use was removed as a matching factor. Axi-cel, axicabtagene ciloleucel; CI, confidence interval; ESS, effective sample size; liso-cel, lisocabtagene maraleucel; N, sample size; NR, not reached

The MAIC results showed no statistically significant difference in the rate of disease progression or mortality (PFS) or the rate of mortality (OS) between liso-cel and axi-cel. In unmatched and unadjusted comparisons, median (95% CI) PFS was similar for liso-cel (4.1 [3.0–6.0] months) versus axi-cel (5.8 [3.4–15.0] months; HR 1.20; 95% CI 0.90–1.59; *P* = 0.219; Table 4). In the initial analysis, the median PFS for liso-cel increased to 6.3 months (ESS = 40.0; 95% CI 3.0–not reached [NR]), and the HR remained similar for liso-cel versus axi-cel (HR 0.95; 95% CI 0.58–1.57; *P* = 0.847; Fig. 1). In SA1 (ESS = 151.4) and SA2 (ESS = 98.9), the median (95% CI) PFS for liso-cel was 3.5 (3.0–5.9) months and 5.0 (3.0–9.2) months, respectively, and HR (95% CI) remained similar between treatments (SA1, 1.30 [0.96–1.77]; *P* = 0.095; SA2, 1.16 [0.81–1.66]; *P* = 0.408).

**Table 4:** Summary of PFS and OS comparisons between the TRANSCEND and ZUMA-1 studies

	ZUMA-1 (axi-cel)		TRANSCEND (liso-cel)		Liso-cel versus axi-cel	
	*N* (%)	Median (95% CI), mo	*N*/ESS	Median (95% CI), mo	HR (95% CI)	*P*
PFS						
Naïve comparison	101	5.8 (3.4–15.0)*	256	4.1 (3.0–6.0)	1.20 (0.90–1.59)	0.219
Initial analysis			40.0	6.3 (3.0–NR)	0.95 (0.58–1.57)	0.847
Sensitivity analysis 1			151.4	3.5 (3.0–5.9)	1.30 (0.96–1.77)	0.095
Sensitivity analysis 2			98.9	5.0 (3.0–9.2)	1.16 (0.81–1.66)	0.408
OS						
Naïve comparison	101	NR (12.8–NR)*	256	21.1 (13.3–NR)	1.13 (0.81–1.58)	0.457
Initial analysis			38.3	NR (11.6–NR)	0.81 (0.44–1.49)	0.506
Sensitivity analysis 1			152.6	19.9 (12.1–NR)	1.15 (0.80–1.65)	0.454
Sensitivity analysis 2			98.9	21.1 (14.4–NR)	1.04 (0.70–1.56)	0.838

Axi-cel, axicabtagene ciloleucel; CI, confidence interval; ESS, effective sample size; HR, hazard ratio; liso-cel, lisocabtagene maraleucel; N, sample size; NR, not reached; OS, overall survival; PFS, progression-free survival

^*^The median was obtained from pseudo-individual patient data based on a digitized Kaplan–Meier curve [16]

**Fig. 2:**
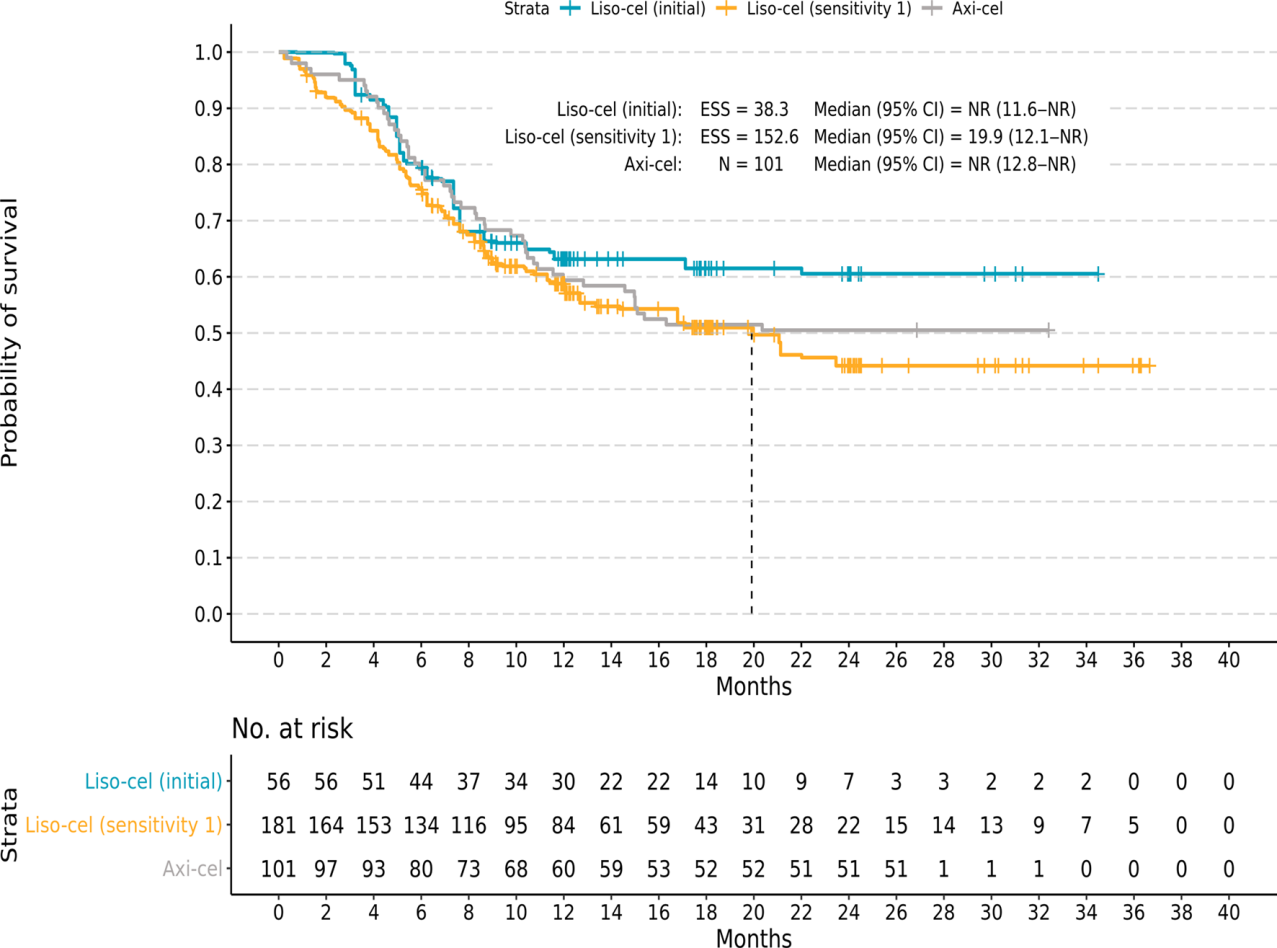
Kaplan–Meier curves for overall survival. Kaplan–Meier curves are shown for the initial and sensitivity analysis comparisons with liso-cel versus axi-cel for infused patients. Kaplan–Meier curves for the initial analysis, which matched and adjusted for 10 factors, including bridging therapy use, demonstrated similar cumulative probabilities of OS for liso-cel and axi-cel. Similar results were observed for sensitivity analysis 1, which was the same as the initial analysis except that bridging therapy use was removed as a matching factor. Axi-cel, axicabtagene ciloleucel; CI, confidence interval; ESS, effective sample size; liso-cel, lisocabtagene maraleucel; N, sample size; NR, not reached

In unmatched and unadjusted comparisons, median (95% CI) OS was 21.1 (13.3–NR) months for liso-cel, with a median follow-up of 17.5 months, and was NR (12.8–NR) for axi-cel and median follow-up was not reported for the OS endpoint, with no significant difference based on HR (HR 1.13; 95% CI 0.81–1.58; *P* = 0.457; Table 4). In the initial analysis, the median OS for liso-cel was NR (ESS = 38.3; 95% CI 11.6–NR), and the survival rate remained similar for liso-cel and axi-cel (HR 0.81; 95% CI 0.44–1.49; *P* = 0.506; Fig. 2). In SA1 (ESS = 152.6) and SA2 (ESS = 98.9), the median (95% CI) OS for liso-cel was 19.9 (12.1–NR) months and 21.1 (14.4–NR) months, respectively, and the HR (95% CI) remained similar (SA1, 1.15 [0.80–1.65]; *P* = 0.454; SA2, 1.04 [0.70–1.56]; *P* = 0.838).

### Safety outcomes

Safety analyses were conducted using the LBCL-treated set from TRANSCEND and the phase 1/2 safety analysis set from ZUMA-1. In the initial safety analysis that matched and adjusted for 9 factors, including bridging therapy use, liso-cel generally had a lower probability of AEs of interest than axi-cel (Table 5). Specifically, liso-cel was associated with a statistically significantly lower probability of all-grade and grade ≥ 3 CRS, all-grade and grade ≥ 3 NEs per study protocol, all-grade and grade ≥ 3 encephalopathy and all-grade aphasia that were reported as NEs, grade ≥ 3 infections, grade ≥ 3 prolonged anemia, grade ≥ 3 prolonged thrombocytopenia, and all-grade hypogammaglobulinemia. Similar findings were reported in the sensitivity analysis that adjusted for all of the same factors except bridging therapy use. Differences were that the lower probability of grade ≥ 3 prolonged neutropenia with liso-cel was now statistically significant, and the following were no longer statistically significantly lower with liso-cel: grade ≥ 3 prolonged anemia, grade ≥ 3 prolonged thrombocytopenia, and all-grade hypogammaglobulinemia.

**Table 5:** Summary of MAIC results for safety outcomes of liso-cel versus axi-cel

Safety outcome, scenario	ZUMA-1 (axi-cel) phase 1/2 safety analysis set		TRANSCEND (liso-cel) LBCL-treated set		Liso-cel versus axi-cel	
	*N*	Event rate, %	*N*/ESS	Event rate, %	OR (95% CI)	*P*
CRS, per Lee 2014 criteria						
All grade						
Naïve	108	92.6	269	42.0	0.06 (0.03–0.12)	< 0.001
Initial			63.0	26.6	0.03 (0.01–0.07)	< 0.001
Sensitivity			209.9	42.9	0.06 (0.03–0.13)	< 0.001
Grade ≥ 3						
Naïve	108	11.1	269	2.2	0.18 (0.07–0.50)	0.001
Initial			63.0	1.0	0.08 (0.01–0.67)	0.019
Sensitivity			209.9	2.0	0.16 (0.06–0.47)	0.001
NE, study-specific						
All grade						
Naïve	108	66.7	269	29.7	0.21 (0.13–0.34)	< 0.001
Initial			63.0	24.6	0.16 (0.08–0.33)	< 0.001
Sensitivity			209.9	29.8	0.21 (0.13–0.35)	< 0.001
Grade ≥ 3						
Naïve	108	32.4	269	10.0	0.23 (0.13–0.41)	< 0.001
Initial			63.0	2.5	0.05 (0.02–0.15)	< 0.001
Sensitivity			209.9	8.7	0.20 (0.11–0.37)	< 0.001
NE of encephalopathy, group term						
All grade						
Naïve	108	37.0	269	21.2	0.46 (0.28–0.75)	0.002
Initial			63.0	19.8	0.42 (0.19–0.91)	0.028
Sensitivity			209.9	20.4	0.44 (0.26–0.73)	0.002
Grade ≥ 3						
Naïve	108	23.1	269	6.7	0.24 (0.12–0.46)	< 0.001
Initial			63.0	1.5	0.05 (0.01–0.18)	< 0.001
Sensitivity			209.9	5.6	0.20 (0.09–0.41)	< 0.001
NE of aphasia, group term						
All grade						
Naïve	108	17.6	269	9.7	0.50 (0.26–0.95)	0.034
Initial			63.0	7.2	0.36 (0.13–1.00)	0.049
Sensitivity			209.9	9.7	0.51 (0.26–1.00)	0.049
Grade ≥ 3						
Naïve	108	7.4	269	1.9	0.24 (0.08–0.74)	0.014
Initial*			–	–	–	–
Sensitivity			209.9	1.5	0.19 (0.06–0.61)	0.005
Infections, any pathogens, per infections and infestations SOC						
Grade ≥ 3						
Naïve	108	26.0	269	12.3	0.40 (0.23–0.70)	0.001
Initial			63.0	6.3	0.19 (0.07–0.49)	0.001
Sensitivity			209.9	11.2	0.36 (0.20–0.65)	0.001
Prolonged anemia, reported as AE						
Grade ≥ 3						
Naïve	108	10.2	269	5.9	0.56 (0.25–1.24)	0.153
Initial			63.0	0.4	0.04 (0.00–0.32)	0.002
Sensitivity			209.9	4.9	0.46 (0.20–1.05)	0.064
Prolonged neutropenia, reported as AE						
Grade ≥ 3						
Naïve	108	25.9	269	14.1	0.47 (0.27–0.82)	0.007
Initial			63.0	13.6	0.45 (0.19–1.07)	0.072
Sensitivity			209.9	15.3	0.52 (0.29–0.91)	0.022
Prolonged thrombocytopenia, reported as AE						
Grade ≥ 3						
Naïve	108	24.1	269	17.5	0.67 (0.39–1.15)	0.143
Initial			63.0	6.9	0.23 (0.10–0.57)	0.001
Sensitivity			209.9	16.3	0.61 (0.35–1.08)	0.090
Hypogammaglobulinemia,^†^ group term						
All grade						
Naïve	108	16.0	269	13.8	0.84 (0.45–1.56)	0.575
Initial			63.0	6.6	0.37 (0.14–0.97)	0.043
Sensitivity			209.9	11.9	0.71 (0.37–1.37)	0.311

TEAEs are reported unless otherwise specified

AE, adverse event; axi-cel, axicabtagene ciloleucel; CI, confidence interval; CRS, cytokine release syndrome; ESS, effective sample size; LBCL, large B cell lymphoma; liso-cel, lisocabtagene maraleucel; MAIC, matching-adjusted indirect comparison; N, sample size; NA, not applicable; NE, neurological event; OR, odds ratio; SOC, system organ class; TEAE, treatment-emergent adverse event

^*^MAICs not possible because all patients with grade ≥ 3 events would have been excluded during matching

^†^Represents TEAE assessed by investigators

#### CRS and NEs

Unmatched and unadjusted rates of CRS were lower for liso-cel versus axi-cel, with a statistically significantly lower probability of all-grade (OR 0.06; 95% CI 0.03–0.12; *P* < 0.001) and grade ≥ 3 (OR 0.18; 95% CI 0.07–0.50; *P* = 0.001) CRS with liso-cel (Table 5). After matching and adjusting for 9 clinical factors, the probability of all-grade (OR 0.03; 95% CI 0.01–0.07; *P* < 0.001) and grade ≥ 3 (OR 0.08; 95% CI 0.01–0.67; *P* = 0.019) CRS remained statistically significantly lower with liso-cel versus axi-cel. In the sensitivity analysis, the probability of all-grade (OR 0.06; 95% CI 0.03–0.13; *P* < 0.001) and grade ≥ 3 (OR 0.16; 95% CI 0.06–0.47; *P* = 0.001) CRS remained significantly lower with liso-cel versus axi-cel.

Unmatched and unadjusted rates of study-specific NEs were lower for liso-cel versus axi-cel, with statistically significantly lower probability of all-grade (OR 0.21; 95% CI 0.13–0.34; *P* < 0.001) and grade ≥ 3 (OR 0.23; 95% CI 0.13–0.41; *P* < 0.001) NEs with liso-cel (Table 5). After matching and adjusting, the probability of all-grade (OR 0.16; 95% CI 0.08–0.33; *P* < 0.001) and grade ≥ 3 (OR 0.05; 95% CI 0.02–0.15; *P* < 0.001) NEs remained statistically significantly lower with liso-cel versus axi-cel. In the sensitivity analysis, the probability of all-grade (OR 0.21; 95% CI 0.13–0.35; *P* < 0.001) and grade ≥ 3 (OR 0.20; 95% CI 0.11–0.37; *P* < 0.001) NEs remained statistically significantly lower with liso-cel versus axi-cel.

Study-specific NEs of encephalopathy and aphasia were analyzed as group terms. The probability of all-grade and grade ≥ 3 encephalopathy (OR 0.42; 95% CI 0.19–0.91; *P* = 0.028 and OR 0.05; 95% CI 0.01–0.18; *P* < 0.001, respectively) as well as all-grade aphasia (OR 0.36; 95% CI 0.13–1.00; *P* = 0.049) was statistically significantly lower with liso-cel versus axi-cel after matching and adjusting for 9 clinical factors (Table 5). In the sensitivity analysis, the probability of all-grade and grade ≥ 3 encephalopathy (OR 0.44; 95% CI 0.26–0.73; *P* = 0.002 and OR 0.20; 95% CI 0.09–0.41; *P* < 0.001, respectively) as well as all-grade aphasia (OR 0.51; 95% CI 0.26–1.00; *P* = 0.049) remained statistically significantly lower with liso-cel versus axi-cel. Unmatched and unadjusted rates of grade ≥ 3 aphasia were lower with liso-cel (1.9%) versus axi-cel (7.4%); however, after matching on bridging therapy use, all patients with grade ≥ 3 aphasia in TRANSCEND were excluded (ie, rate of 0% for liso-cel). Thus, further adjustments based on patient weights were not estimable. In the sensitivity analysis that did not match on bridging therapy use, the probability of grade ≥ 3 aphasia was statistically significantly lower with liso-cel versus axi-cel (OR 0.19; 95% CI 0.06–0.61; *P* = 0.005).

Use of tocilizumab and corticosteroids to manage CRS and/or study-specific NEs was compared between studies. Notably, for CRS and/or NE management, the overall use of tocilizumab was lower in TRANSCEND than in ZUMA-1 (20% vs 43%, respectively), as was use of corticosteroids (21% vs 27%, respectively).

#### Additional AEs of interest

The probability of grade ≥ 3 infections was statistically significantly lower with liso-cel versus axi-cel before (OR 0.40; 95% CI 0.23–0.70; *P* = 0.001) and after (OR 0.19; 95% CI 0.07–0.49; *P* = 0.001) matching and adjusting (Table 5). Unmatched and unadjusted rates of all-grade hypogammaglobulinemia were similar for liso-cel and axi-cel, with no statistically significant difference in the probability of the event between treatments. After matching and adjusting, the probability of all-grade hypogammaglobulinemia was statistically significantly lower for liso-cel versus axi-cel (OR 0.37; 95% CI 0.14–0.97; *P* = 0.043). In the sensitivity analysis, the probability of grade ≥ 3 infections remained significantly lower with liso-cel versus axi-cel (OR 0.36; 95% CI 0.20–0.65; *P* = 0.001), while the probability of all-grade hypogammaglobulinemia was no longer statistically significantly lower for liso-cel (OR 0.71; 95% CI 0.37–1.37; *P* = 0.311).

An unadjusted comparison between TRANSCEND and ZUMA-1 data was conducted for prolonged cytopenia (grade ≥ 3 anemia, neutropenia, or thrombocytopenia not resolved by Day 29 [TRANSCEND] or by Day 30 [ZUMA-1]). Grade ≥ 3 prolonged cytopenia occurred in 38% of patients in ZUMA-1 (per investigator assessment of AEs) and in 37% of patients in TRANSCEND (per laboratory assessment).

When comparing investigator-reported AEs of grade ≥ 3 prolonged anemia, neutropenia, and thrombocytopenia (at Day 29 for TRANSCEND or Day 30 for ZUMA-1), the probability of an event was lower for liso-cel versus axi-cel. Unmatched and unadjusted probability of AEs of grade ≥ 3 prolonged anemia was not statistically significantly lower for liso-cel versus axi-cel (OR 0.56; 95% CI 0.25–1.24; *P* = 0.153). After matching and adjusting, the probability of grade ≥ 3 prolonged anemia was statistically significantly lower for liso-cel versus axi-cel in the initial analysis (OR 0.04; 95% CI 0.00–0.32; *P* = 0.002) but not the sensitivity analysis (OR 0.46; 95% CI 0.20–1.05; *P* = 0.064).

Unmatched and unadjusted probability of AEs of grade ≥ 3 prolonged neutropenia was statistically significantly lower for liso-cel versus axi-cel (OR 0.47; 95% CI 0.27–0.82; *P* = 0.007). After matching and adjusting, the probability of grade ≥ 3 prolonged neutropenia was no longer statistically significantly lower for liso-cel versus axi-cel in the initial analysis (OR 0.45; 95% CI 0.19–1.07; *P* = 0.072) but was in the sensitivity analysis (OR 0.52; 95% CI 0.29–0.91; *P* = 0.022).

Unmatched and unadjusted probability of AEs of grade ≥ 3 prolonged thrombocytopenia was not statistically significantly different for liso-cel versus axi-cel (OR 0.67; 95% CI 0.39–1.15; *P* = 0.143). After matching and adjusting, the probability of grade ≥ 3 prolonged thrombocytopenia was statistically significantly lower for liso-cel versus axi-cel in the initial analysis (OR 0.23; 95% CI 0.10–0.57; *P* = 0.001) but not the sensitivity analysis (OR 0.61; 95% CI 0.35–1.08; *P* = 0.090).

## Discussion

Results from this MAIC of liso-cel versus axi-cel showed that liso-cel had comparable efficacy and a favorable safety profile compared with axi-cel. The feasibility assessment identified 18 clinical factors reported in both TRANSCEND and ZUMA-1 that were available for adjustment and determined that imbalances between studies were mostly small to moderate and suitable for MAIC-based methodology. Therefore, it was determined that MAIC would achieve reductions in between-study imbalances without significant loss in ESS. MAICs were deemed feasible for 4 efficacy outcomes and 9 safety outcomes.

The initial analysis that matched and adjusted for 10 clinical factors (including bridging therapy use) showed that liso-cel and axi-cel have similar efficacy measured as ORR (OR 1.40 [95% CI 0.56–3.49]), CRR (OR 1.21 [95% CI 0.56–2.64]), PFS (HR 0.95 [95% CI 0.58–1.57]), and OS (HR 0.81 [95% CI 0.44–1.49]) as axi-cel. Similar results were observed in sensitivity analyses that excluded bridging therapy use as a matching factor and adjusted for additional potentially prognostic clinical factors.

Adjusted safety outcomes showed statistically significantly lower rates of CRS, study-specific NEs (including those of encephalopathy and aphasia), and grade ≥ 3 infections with liso-cel versus axi-cel. There are several factors that could contribute to the more favorable safety profile observed with liso-cel, including the 4-1BB co-stimulatory domain, which is reportedly associated with a lower incidence of CRS and NEs than CD28-containing construct [30]. In addition, as variability in total and CD8^+^ CAR T cells has been associated with increased toxicity, the administration of liso-cel at a defined composition with consistent total and relative doses of CD8^+^:CD4^+^ CAR^+^ T cells may also contribute to reduced toxicity [31, 32].

Furthermore, as reported per study, tocilizumab and corticosteroid use for management of CRS and/or NEs was also lower in TRANSCEND than in ZUMA-1. Although these interventions could potentially impact the severity of CRS and/or NEs, they would not affect the overall incidence of all-grade CRS or NEs, as they are administered after the onset of the event. As use of tocilizumab and corticosteroids was lower in TRANSCEND, this would not account for the lower incidence of all-grade and lower probability of grade ≥ 3 events associated with liso-cel. Additionally, it is possible that frequency of use of tocilizumab and corticosteroids may have changed over the course of the studies as physicians gained experience treating and managing patients with these AEs. Although we were unable to evaluate this in the analysis, changes in the rates of grade ≥ 3 CRS or NEs over time, as reported per study from early interim analyses to primary data cuts, appear to have been minimal (TRANSCEND interim analysis based on 91 patients versus primary data cut based on 269 patients: grade ≥ 3 CRS was 1% versus 2%, respectively, and grade ≥ 3 NE was 12% versus 10%, respectively; ZUMA-1 interim analysis based on 51 patients versus primary data cut based on 101 patients: grade ≥ 3 CRS was 20% versus 13%, respectively, and grade ≥ 3 NE was 29% versus 28%, respectively) [7, 8, 33, 34].

Our feasibility assessment identified the use of bridging therapy as a notable difference between TRANSCEND and ZUMA-1. ZUMA-1 did not permit bridging therapy, whereas TRANSCEND allowed bridging therapy use at the discretion of the treating clinicians. Use of bridging therapy could potentially be associated with more aggressive disease or with differences between products in the manufacturing time requirement. To explore the potential impact of bridging therapy use, the initial MAIC analyses matched on bridging therapy use and additional sensitivity analyses were conducted without matching on bridging therapy use. No statistically significant differences in efficacy were observed between liso-cel and axi-cel in either analysis.

A similar MAIC was recently conducted to estimate treatment effects with axi-cel versus tisagenlecleucel based on data from ZUMA-1 and JULIET [35]. The study reported that axi-cel had a higher ORR and CRR, longer OS, higher rates of grade 1/2 CRS, and similar rates of grade ≥ 3 CRS and NEs compared with tisagenlecleucel. In the ZUMA-1 and JULIET comparison, matching for bridging therapy use was not possible because 92% of patients in JULIET received bridging therapy and it was not allowed in ZUMA-1 [9, 35]. In contrast, our study was able to match patients for bridging therapy use owing to the large number of patients in TRANSCEND contributing to the IPD (*N* = 269) and the fact that 110 patients remained after removing the 59% of patients in TRANSCEND who received bridging therapy [7]. Thus, a sufficient number of patients was available to conduct analyses in patients who did not receive bridging therapy (110 from TRANSCEND and 108 from ZUMA-1).

A notable strength of this study was the rigorous, multifaceted process undertaken to identify and rank-order clinically relevant factors. In addition, multiple publications were evaluated to identify the most compatible cohorts from the comparator study to those available in TRANSCEND [8, 16–19]. Alignments on variable and outcome measure definitions through recalculations or recategorizations with TRANSCEND data were made and documented wherever possible, which further helped to reduce bias in downstream estimates. Finally, sensitivity analyses were conducted to test the robustness of results presented as initial analyses within this study.

Limitations to the current analysis include those inherent to an unanchored MAIC. Despite the methodological rigor, there may be clinical factors unaccounted for in this process. Some clinical factors identified and deemed important through this process were not reported for ZUMA-1 in a format conducive for comparison or alignment with TRANSCEND, such as lactate dehydrogenase, C-reactive protein, and best response to any prior therapy. Thus, some imbalance remained between variables after matching and adjusting that could have influenced point estimates in an unknown direction and magnitude.

As our analysis found comparable efficacy and a more favorable safety profile with liso-cel compared with axi-cel, it could have implications on clinical practice. The lower incidence of CRS and later onset allows outpatient administration at centers with adequate infrastructure; this may decrease hospital utilization [7, 8, 36]. A more favorable safety profile would be advantageous in all patients as well as those with comorbidities. In addition to this indirect treatment comparison, real-world evidence comparing the safety and efficacy of CAR T cell therapies could provide additional insights to help inform clinical decisions. Although analyses of real-world data for liso-cel are not currently available, real-world evidence for axi-cel and tisagenlecleucel has shown that efficacy and safety outcomes in clinical practice are consistent with those reported in clinical studies [37, 38]. In a post-marketing study of axi-cel in 1001 patients with R/R LBCL and ≥ 6 months of follow-up, the best ORR was 70% and CRR was 53%; CRS by Lee 2014 criteria of any grade was reported in 83% of patients (grade ≥ 3: 10%) and NEs (defined as immune effector cell-associated neurotoxicity syndrome) of any grade were reported in 57% (grade ≥ 3: 26%) [38]. These outcomes are consistent with those reported in ZUMA-1 (ORR: 82%; CRR: 54%; any-grade CRS: 93%; grade ≥ 3 CRS: 13%; any-grade NEs: 64%; grade ≥ 3 NEs: 28%) [8].

## Conclusions

In summary, we used unanchored MAICs that leveraged IPD from TRANSCEND and summary-level data from ZUMA-1 to derive indirect comparisons while accounting for between-study differences in eligibility criteria and baseline characteristics. Results were similar for initial and sensitivity analyses, supporting the robustness of the findings. Overall, after matching and adjusting for important clinical prognostic factors and treatment-effect modifiers, liso-cel demonstrated similar efficacy and a favorable safety profile compared with axi-cel.

## Supplementary Information

**Additional file 1**. Supplementary Appendix.

## Data Availability

Bristol-Myers Squibb policy on data sharing may be found at https://www.bms.com/researchers-and-partners/independent-research/data-sharing-request-process.html.

## Abbreviations

AE: Adverse event
ALC: Absolute lymphocyte count
allo-HSCT: Allogeneic hematopoietic stem cell transplantation
auto-HSCT: Autologous hematopoietic stem cell transplantation
axi-cel: Axicabtagene ciloleucel
CAR: Chimeric antigen receptor
CI: Confidence interval
CNS: Central nervous system
CRR: Complete response rate
CrCl: Creatinine clearance
CRS: Cytokine release syndrome
DLBCL: Diffuse large B cell lymphoma
ECOG PS: Eastern Cooperative Oncology Group performance status
ESS: Effective sample size
FL: Follicular lymphoma
FL3B: Follicular lymphoma grade 3B
HGBCL: High-grade B cell lymphoma
HR: Hazard ratio
IPD: Individual patient data
IPI: International Prognostic Index
IRC: Independent review committee
LBCL: Large B cell lymphoma
LDC: Lymphodepleting chemotherapy
liso-cel: Lisocabtagene maraleucel
LVEF: Left ventricular ejection fraction
MAIC: Matching-adjusted indirect comparison
mITT: Modified intention to treat
N: Sample size
NA: Not applicable
ND/PD SOC: Nervous system disorder/psychiatric disorder system organ class
NE: Neurological event
NHL: Non-Hodgkin lymphoma
NOS: Not otherwise specified
NR: Not reached
OR: Odds ratio
ORR: Objective response rate
OS: Overall survival
PET: Positron emission tomography
PFS: Progression-free survival
PMBCL: Primary mediastinal B cell lymphoma
R/R: Relapsed or refractory
SA1: Sensitivity analysis 1
SA2: Sensitivity analysis 2
SD: Standard deviation
SMD: Standard mean difference
SPD: Sum of the product of perpendicular diameters
Stat: Statistic
tFL: Transformed follicular lymphoma
tiNHL: Transformed indolent non-Hodgkin lymphoma

## References

[1] Bray F, Ferlay J, Soerjomataram I, Siegel RL, Torre LA, Jemal A (2018). Global cancer statistics 2018: GLOBOCAN estimates of incidence and mortality worldwide for 36 cancers in 185 countries. CA Cancer J Clin.

[2] Hunt KE, Reichard KK (2008). Diffuse large B-cell lymphoma. Arch Pathol Lab Med.

[3] Martelli M, Ferreri AJ, Agostinelli C, Di Rocco A, Pfreundschuh M, Pileri SA (2013). Diffuse large B-cell lymphoma. Crit Rev Oncol Hematol.

[4] Crump M, Neelapu SS, Farooq U, Van Den Neste E, Kuruvilla J, Westin J (2017). Outcomes in refractory diffuse large B-cell lymphoma: results from the international SCHOLAR-1 study. Blood.

[5] Gisselbrecht C, Van Den Neste E (2018). How I manage patients with relapsed/refractory diffuse large B cell lymphoma. Br J Haematol.

[6] Beham-Schmid C (2017). Aggressive lymphoma 2016: revision of the WHO classification. Memo.

[7] Abramson JS, Palomba ML, Gordon LI, Lunning MA, Wang M, Arnason J (2020). Lisocabtagene maraleucel for patients with relapsed or refractory large B-cell lymphomas (TRANSCEND NHL 001): a multicentre seamless design study. Lancet.

[8] Neelapu SS, Locke FL, Bartlett NL, Lekakis LJ, Miklos DB, Jacobson CA (2017). Axicabtagene ciloleucel CAR T-cell therapy in refractory large B-cell lymphoma. N Engl J Med.

[9] Schuster SJ, Bishop MR, Tam CS, Waller EK, Borchmann P, McGuirk JP (2019). Tisagenlecleucel in adult relapsed or refractory diffuse large B-cell lymphoma. N Engl J Med.

[10] YESCARTA (axicabtagene ciloleucel). [Package insert]. Santa Monica: Kite Pharma, Inc.; 2020.

[11] KYMRIAH (tisagenlecleucel). [Package insert]. East Hanover: Novartis Pharmaceuticals Corporation; 2018.

[12] European Medicines Agency. Yescarta (axicabtagene ciloleucel). 2019.

[13] European Medicines Agency. Kymriah (tisagenlecleucel). 2019.

[14] BREYANZI (lisocabtagene maraleucel). [Prescribing information]. Princeton: Bristol Myers Squibb; 2021.

[15] Maude SL, Frey N, Shaw PA, Aplenc R, Barrett DM, Bunin NJ (2014). Chimeric antigen receptor T cells for sustained remissions in leukemia. N Engl J Med.

[16] Locke FL, Ghobadi A, Jacobson CA, Miklos DB, Lekakis LJ, Oluwole OO (2019). Long-term safety and activity of axicabtagene ciloleucel in refractory large B-cell lymphoma (ZUMA-1): a single-arm, multicentre, phase 1–2 trial. Lancet Oncol.

[17] US Food and Drug Administration. YESCARTA (axicbatagene ciloleucel). BLA Clinical Review Memorandum. https://www.fda.gov/media/109149/download. Accessed 5 Oct 2020.

[18] YESCARTA (axicbatagene ciloleucel) [Summary of product characteristics]. Amsterdam: Kite Pharma EU B.V.; 2018.

[19] European Medicines Agency. YESCARTA (axicbatagene ciloleucel). European Public Assessment Report. https://www.ema.europa.eu/en/medicines/human/EPAR/yescarta. Accessed 5 Oct 2020.

[20] Zhang J, Li J, Ma Q, Yang H, Signorovitch J, Wu E (2020). A review of two regulatory approved anti-CD19 CAR T-cell therapies in diffuse large B-cell lymphoma: why are indirect treatment comparisons not feasible?. Adv Ther.

[21] Cheson BD, Pfistner B, Juweid ME, Gascoyne RD, Specht L, Horning SJ (2007). Revised response criteria for malignant lymphoma. J Clin Oncol.

[22] Cheson BD, Fisher RI, Barrington SF, Cavalli F, Schwartz LH, Zucca E (2014). Recommendations for initial evaluation, staging, and response assessment of Hodgkin and non-Hodgkin lymphoma: the Lugano classification. J Clin Oncol.

[23] Lee DW, Gardner R, Porter DL, Louis CU, Ahmed N, Jensen M (2014). Current concepts in the diagnosis and management of cytokine release syndrome. Blood.

[24] Phillippo DM, Ades AE, Dias S, Palmer S, Abrams KR, Welton NJ (2018). Methods for population-adjusted indirect comparisons in health technology appraisal. Med Decis Making.

[25] Altmann A, Toloşi L, Sander O, Lengauer T (2010). Permutation importance: a corrected feature importance measure. Bioinformatics.

[26] Breiman L (2001). Random forests. Mach Learn.

[27] Ishwaran H, Kogalur UB, Blackstone EH, Lauer MS (2008). Random survival forests. Ann Appl Stat.

[28] Guyot P, Ades AE, Ouwens MJ, Welton NJ (2012). Enhanced secondary analysis of survival data: reconstructing the data from published Kaplan-Meier survival curves. BMC Med Res Methodol.

[29] Austin PC (2009). Balance diagnostics for comparing the distribution of baseline covariates between treatment groups in propensity-score matched samples. Stat Med.

[30] Lu P, Lu X-A, Zhang X, Zhang J, Zhou X, Qi F (2018). Which is better in CD19 CAR-T treatment of r/r B-ALL, CD28 or 4-1BB? A parallel trial under the same manufacturing process. J Clin Oncol.

[31] Hay KA, Hanafi LA, Li D, Gust J, Liles WC, Wurfel MM (2017). Kinetics and biomarkers of severe cytokine release syndrome after CD19 chimeric antigen receptor-modified T-cell therapy. Blood.

[32] Turtle CJ, Hanafi LA, Berger C, Hudecek M, Pender B, Robinson E (2016). Immunotherapy of non-Hodgkin's lymphoma with a defined ratio of CD8+ and CD4+ CD19-specific chimeric antigen receptor-modified T cells. Sci Transl Med..

[33] Neelapu SS, Locke FL, Bartlett NL, Lekakis L, Miklos D, Jacobson CA (2016). Kte-C19 (anti-CD19 CAR T cells) induces complete remissions in patients with refractory diffuse large B-cell lymphoma (DLBCL): results from the pivotal phase 2 Zuma-1. Blood.

[34] Abramson JS, Gordon LI, Palomba ML, Lunning MA, Arnason JE, Forero-Torres A (2018). Updated safety and long term clinical outcomes in TRANSCEND NHL 001, pivotal trial of lisocabtagene maraleucel (JCAR017) in R/R aggressive NHL. J Clin Oncol.

[35] Oluwole OO, Jansen JP, Lin VW, Chan K, Keeping S, Navale L (2020). Comparing efficacy, safety, and preinfusion period of axicabtagene ciloleucel versus tisagenlecleucel in relapsed/refractory large B cell lymphoma. Biol Blood Marrow Transplant.

[36] Abramson JS, Siddiqi T, Garcia J, Dehner C, Kim Y, Nguyen A (2021). Cytokine release syndrome and neurological event costs in lisocabtagene maraleucel-treated patients in the TRANSCEND NHL 001 trial. Blood Adv.

[37] Le Gouill S, Bachy E, Di Blasi R, Carton G, Beauvais D, Le Bras F, et al. First results of DLBCL patients treated with CAR-T cells and enrolled in DESCAR-T registry, a French real-life database for CAR-T cells in hematologic malignancies. European Hematology Association; Virtual 2021.

[38] Jacobson CA, Locke FL, Hu Z-H, Siddiqi T, Ahmed S, Ghobadi A (2021). Real-world evidence of axicabtagene ciloleucel (Axi-cel) for the treatment of large B-cell lymphoma (LBCL) in the United States (US). J Clin Oncol.

[39] Swerdlow SH, Campo E, Harris NL, Jaffe ES, Pileri SA, Stein H (2008). WHO classification of tumours of haematopoietic and lymphoid tissues.

