# Supplementary material for: Matching-adjusted indirect treatment comparison of liso-cel versus axi-cel in relapsed or refractory large B cell lymphoma
Source: J Hematol Oncol. 2021 Sep 8;14:140. doi: 10.1186/s13045-021-01144-9 (PMC8425084; doi:10.1186/s13045-021-01144-9)
Supplement: Supplementary file 1 — Additional file 1. Supplementary Appendix. [file 13045_2021_1144_MOESM1_ESM.docx]

**Supplementary Appendix**

**Matching-adjusted indirect treatment comparison of liso-cel vs axi-cel for treatment of relapsed or refractory large B-cell lymphoma**

David G. Maloney, John Kuruvilla, Fei Fei Liu, Ana Kostic, Yeonhee Kim, Ashley Bonner, Yixie Zhang, Christopher P. Fox and Guillaume Cartron

**Table of Contents**

[Methods 3](#_Toc69223906)

[Supplemental Table 1. Clinical factor selection for the initial analysis, SA1, and SA2 of efficacy outcomes for liso-cel– vs axi-cel–infused patients 3](#_Toc69223907)

[Results 6](#_Toc69223908)

[Supplemental Table 2. Comparison of clinical factors and SMDs before and after MAIC for PFS 6](#_Toc69223909)

[Supplemental Table 3. Comparison of clinical factors and SMDs before and after MAIC for CRR 10](#_Toc69223910)

[Supplemental Table 4. Comparison of clinical factors and SMDs before and after MAIC for ORR 15](#_Toc69223911)

[Supplemental Table 5. Comparison of clinical factors and SMDs before and after MAIC for safety analysis 20](#_Toc69223912)

Methods

Supplemental Table 1. Clinical factor selection for the initial analysis, SA1, and SA2 of efficacy outcomes for liso-cel– vs axi-cel–infused patients

|  |  | **ORR** | | | **CRR** | | | **PFS** | | | **OS** | | |
| --- | --- | --- | --- | --- | --- | --- | --- | --- | --- | --- | --- | --- | --- |
|  | **Clinical factor** | **Initial** | **SA1** | **SA2** | **Initial** | **SA1** | **SA2** | **Initial** | **SA1** | **SA2** | **Initial** | **SA1** | **SA2** |
| Factors matched | Bridging therapy | ✓ |  |  | ✓ |  |  | ✓ |  |  | ✓ |  |  |
|  | Disease histology | ✓ | ✓ | ✓ | ✓ | ✓ | ✓ | ✓ | ✓ | ✓ | ✓ | ✓ | ✓ |
|  | ECOG PS | ✓ | ✓ | ✓ | ✓ | ✓ | ✓ | ✓ | ✓ | ✓ | ✓ | ✓ | ✓ |
|  | Secondary CNS involvement | ✓ | ✓ | ✓ | ✓ | ✓ | ✓ | ✓ | ✓ | ✓ | ✓ | ✓ | ✓ |
|  | Prior allo-HSCT | ✓ | ✓ | ✓ | ✓ | ✓ | ✓ | ✓ | ✓ | ✓ | ✓ | ✓ | ✓ |
| Factors adjusted | Tumor burden | ✓ | ✓ | ✓ | ✓ | ✓ | ✓ | ✓ | ✓ | ✓ | ✓ | ✓ | ✓ |
|  | IPI score |  |  | ✓ |  |  | ✓ | ✓ | ✓ | ✓ | ✓ | ✓ | ✓ |
|  | R/R status to last therapy | ✓ | ✓ | ✓ | ✓ | ✓ | ✓ | ✓ | ✓ | ✓ | ✓ | ✓ | ✓ |
|  | Bulky disease |  |  | ✓ | ✓ | ✓ | ✓ | ✓ | ✓ | ✓ | ✓ | ✓ | ✓ |
|  | Age |  |  | ✓ |  |  | ✓ |  |  | ✓ | ✓ | ✓ | ✓ |
|  | Prior auto-HSCT | ✓ | ✓ | ✓ | ✓ | ✓ | ✓ |  |  | ✓ |  |  | ✓ |
|  | Disease stage | ✓ | ✓ | ✓ |  |  | ✓ |  |  | ✓ |  |  | ✓ |
|  | CrCl | ✓ | ✓ | ✓ |  |  | ✓ | ✓ | ✓ | ✓ |  |  | ✓ |
|  | Extranodal disease |  |  | ✓ | ✓ | ✓ | ✓ |  |  | ✓ |  |  | ✓ |
|  | Number of prior therapies |  |  | ✓ |  |  | ✓ |  |  | ✓ |  |  | ✓ |
|  | Sex |  |  | ✓ |  |  | ✓ |  |  | ✓ |  |  | ✓ |
|  | Absolute lymphocyte count |  |  | ✓ |  |  | ✓ |  |  | ✓ |  |  | ✓ |
|  | LVEF |  |  | ✓ |  |  | ✓ |  |  | ✓ |  |  | ✓ |

Allo-HSCT, allogeneic hematopoietic stem cell transplantation; auto-HSCT, autologous hematopoietic stem cell transplantation; axi-cel, axicabtagene ciloleucel; CNS, central nervous system; CRR, complete response rate; CrCl, creatinine clearance; ECOG PS, Eastern Cooperative Oncology Group performance status; IPI, International Prognostic Index; liso-cel, lisocabtagene maraleucel; LVEF, left ventricular ejection fraction; ORR, objective response rate; OS, overall survival; PFS, progression-free survival; R/R, relapsed or refractory; SA1, sensitivity analysis 1; SA2, sensitivity analysis 2.

Results

Supplemental Table 2. Comparison of clinical factors and SMDs before and after MAIC for PFS

| **Clinical factor** | **PFS** | | | | | | | | |
| --- | --- | --- | --- | --- | --- | --- | --- | --- | --- |
|  | **ZUMA-1  (axi-cel) phase 2 mITT set** | **TRANSCEND (liso-cel) LBCL efficacy set** | | | | | | | |
|  |  | **Before MAIC  (naïve)** | | **After MAIC (initial)** | | **After MAIC  (SA1)** | | **After MAIC  (SA2)** | |
| **N/ESS** | **N=101** | **N=256** | | **ESS=40.0** | | **ESS=151.4** | | **ESS=98.9** | |
|  | **Stat.** | **Stat.** | **SMD** | **Stat.** | **SMD** | **Stat.** | **SMD** | **Stat.** | **SMD** |
| Age, y, mean (SD) | 56.3 (12.0) | 60.3 (13.3) | 0.308 | 59.0 (12.3) | 0.022 | 58.2 (13.7) | 0.142 | 56.3 (12.0) | 0.000 |
| Male sex, % | 67.3 | 66.0 | 0.027 | 79.2 | 0.272 | 73.8 | 0.143 | 67.3 | 0.000 |
| IPI score, %* |  |  |  |  |  |  |  |  |  |
| 0–2 | 54.5 | 58.6 | 0.162 | 54.5 | 0.000 | 54.5 | 0.000 | 54.5 | 0.000 |
| 3–4 | 45.5 | 39.8 |  | 45.5 |  | 45.5 |  | 45.5 |  |
| 5 | 0.0 | 0.8 |  | 0.0 |  | 0.0 |  | 0.0 |  |
| Missing | 0.0 | 0.8 |  | 0.0 |  | 0.0 |  | 0.0 |  |
| ECOG PS at screening, % |  |  |  |  |  |  |  |  |  |
| 0 | 41.6 | 40.6 | 0.178 | 41.5 | 0.001 | 38.1 | 0.072 | 41.6 | 0.000 |
| 1 | 58.4 | 57.8 |  | 58.5 |  | 61.9 |  | 58.4 |  |
| 2 | 0.0 | 1.6 |  | 0.0 |  | 0.0 |  | 0.0 |  |
| Disease stage, % |  |  |  |  |  |  |  |  |  |
| I or II | 14.9 | 27.0 | 0.304 | 28.8 | 0.342 | 25.0 | 0.254 | 14.9 | 0.000 |
| III or IV | 85.1 | 72.3 |  | 71.2 |  | 75.0 |  | 85.1 |  |
| Missing | 0.0 | 0.8 |  | 0.0 |  | 0.0 |  | 0.0 |  |
| Tumor burden based on SPD before LDC, cm^2^, mean (SD)^†^ | 50.4 (43.7) | 43.7 (48.1) | 0.142 | 50.4 (44.1) | 0.000 | 50.4 (43.8) | 0.000 | 50.4 (43.9) | 0.000 |
| Secondary CNS disease at time of treatment, % |  |  |  |  |  |  |  |  |  |
| No | 100.0 | 97.7 | 0.219 | 100.0 | 0.000 | 100.0 | 0.000 | 100.0 | 0.000 |
| Yes | 0.0 | 2.3 |  | 0.0 |  | 0.0 |  | 0.0 |  |
|  |  |  |  |  |  |  |  |  |  |
| Extranodal disease, % |  |  |  |  |  |  |  |  |  |
| No | 30.7 | 46.9 | 0.344 | 47.3 | 0.345 | 45.2 | 0.302 | 30.7 | 0.000 |
| Yes | 69.3 | 52.3 |  | 52.7 |  | 54.8 |  | 69.3 |  |
| Missing | 0.0 | 0.8 |  | 0.0 |  | 0.0 |  | 0.0 |  |
| Bulky disease, % |  |  |  |  |  |  |  |  |  |
| No | 83.2 | 87.9 | 0.155 | 83.2 | 0.000 | 83.2 | 0.000 | 83.2 | 0.000 |
| Yes | 16.8 | 11.3 |  | 16.8 |  | 16.8 |  | 16.8 |  |
| Missing | 0.0 | 0.8 |  | 0.0 |  | 0.0 |  | 0.0 |  |
| Disease histology, % |  |  |  |  |  |  |  |  |  |
| DLBCL^‡^ | 76.2 | 71.1 | 0.242 | 76.3 | 0.000 | 76.3 | 0.000 | 76.3 | 0.000 |
| DLBCL tFL | 15.8 | 22.3 |  | 15.8 |  | 15.8 |  | 15.8 |  |
| PMBCL | 7.9 | 5.5 |  | 7.9 |  | 7.9 |  | 7.9 |  |
| FL3B | 0.0 | 1.2 |  | 0.0 |  | 0.0 |  | 0.0 |  |
| No. of lines of prior therapy, %^§^ |  |  |  |  |  |  |  |  |  |
| 1 | 3.0 | 0.8 | 0.179 | 1.1 | 0.348 | 0.7 | 0.175 | 3.0 | 0.000 |
| 2 | 27.7 | 25.0 |  | 43.5 |  | 30.0 |  | 27.7 |  |
| ≥3 | 69.3 | 73.8 |  | 55.4 |  | 69.3 |  | 69.3 |  |
| Missing | 0.0 | 0.4 |  | 0.0 |  | 0.0 |  | 0.0 |  |
| Prior allo-HSCT, % | 0.0 | 2.7 | 0.237 | 0.0 | 0.000 | 0.0 | 0.000 | 0.0 | 0.000 |
| Prior auto-HSCT, % | 24.8 | 33.2 | 0.186 | 34.9 | 0.221 | 27.1 | 0.052 | 24.8 | 0.000 |
| Bridging therapy, % |  |  |  |  |  |  |  |  |  |
| No | 100.0 | 41.4 | 1.682 | 100.0 | 0.000 | 35.2 | 1.921 | 35.9 | 1.889 |
| Yes | 0.0 | 58.6 |  | 0.0 |  | 64.8 |  | 64.1 |  |
|  |  |  |  |  |  |  |  |  |  |
| R/R to last therapy, %^¶^ |  |  |  |  |  |  |  |  |  |
| Relapsed | 20.8 | 35.9 | 0.359 | 20.8 | 0.000 | 20.8 | 0.000 | 20.8 | 0.000 |
| Refractory | 79.2 | 61.7 |  | 79.2 |  | 79.2 |  | 79.2 |  |
| Missing | 0.0 | 2.3 |  | 0.0 |  | 0.0 |  | 0.0 |  |
| CrCl before LDC, %^#^ |  |  |  |  |  |  |  |  |  |
| <60 mL/min | 0.0 | 19.1 | 0.688 | 0.0 | 0.000 | 0.0 | 0.000 | 0.0 | 0.000 |
| $\geq$60 mL/min | 100.0 | 80.9 |  | 100.0 |  | 100.0 |  | 100.0 |  |
| LVEF at screening, %^#^ |  |  |  |  |  |  |  |  |  |
| <50% | 0.0 | 5.1 | 0.327 | 5.9 | 0.354 | 4.5 | 0.306 | 0.0 | 0.000 |
| ≥50% | 100.0 | 94.9 |  | 94.1 |  | 95.5 |  | 100.0 |  |
| Pre-leukapheresis ALC (10^9^/L), %^#^ |  |  |  |  |  |  |  |  |  |
| <0.1 | 0.0 | 0.4 | 0.091 | 0.0 | 0.000 | 0.2 | 0.063 | 0.0 | 0.000 |
| ≥0.1 | 100.0 | 94.1 |  | 93.0 |  | 92.6 |  | 100.0 |  |
| Missing | 0.0 | 5.5 |  | 7.0 |  | 7.3 |  | 0.0 |  |
| Statistics, % |  |  |  |  |  |  |  |  |  |
| Factors with SMD <0.2 | NA | 44.4 | NA | 61.1 | NA | 77.8 | NA | 94.4 | NA |
| Factors with SMD <0.1 | NA | 11.1 | NA | 61.1 | NA | 61.1 | NA | 94.4 | NA |

ALC, absolute lymphocyte count; allo-HSCT, allogeneic hematopoietic stem cell transplantation; auto-HSCT, autologous hematopoietic stem cell transplantation; axi-cel, axicabtagene ciloleucel; CNS, central nervous system; CrCl, creatinine clearance; DLBCL, diffuse large B-cell lymphoma; ECOG PS, Eastern Cooperative Oncology Group performance status; ESS, effective sample size; FL3B, follicular lymphoma grade 3B; HGBCL, high-grade B-cell lymphoma; IPI, International Prognostic Index; LBCL, large B-cell lymphoma; liso-cel, lisocabtagene maraleucel; LDC, lymphodepleting chemotherapy; LVEF, left ventricular ejection fraction; MAIC, matching-adjusted indirect comparison; mITT, modified intention to treat; N, sample size; NA, not applicable; NOS, not otherwise specified; PFS, progression-free survival; PMBCL, primary mediastinal B-cell lymphoma; R/R, relapsed or refractory; SA1, sensitivity analysis 1; SA2, sensitivity analysis 2; SD, standard deviation; SMD, standard mean difference; SPD, sum of the product of perpendicular diameters; Stat., statistic; tFL, transformed follicular lymphoma; tiNHL, transformed indolent non-Hodgkin lymphoma.

*Per ZUMA-1 categorization.

^†^Per investigator assessment.

^‡^Includes DLBCL NOS, HGBCL, and tiNHL for TRANSCEND; includes DLBCL NOS and HGBCL for ZUMA-1.

^§^Per ZUMA-1, salvage chemotherapy and auto-HSCT were considered separate regimens.

^¶^Per ZUMA-1, refractory was defined as best response to last therapy of progressive disease or stable disease and relapsed defined as best response to last therapy of partial response or complete response.

^#^Per ZUMA-1 eligibility criteria, all patients enrolled in ZUMA-1 had CrCl ≥60 mL/min before LDC, LVEF ≥50% at screening, and pre-leukapheresis ALC ≥0.1×10^9^/L.

Supplemental Table 3. Comparison of clinical factors and SMDs before and after MAIC for CRR

| **Clinical factor** | **CRR** | | | | | | | | |
| --- | --- | --- | --- | --- | --- | --- | --- | --- | --- |
|  | **ZUMA-1 (axi-cel) phase 2 mITT set** | **TRANSCEND (liso-cel) LBCL efficacy set** | | | | | | | |
|  |  | **Before MAIC  (naïve)** | | **After MAIC (initial)** | | **After MAIC  (SA1)** | | **After MAIC  (SA2)** | |
| **N/ESS** | **N=101** | **N=256** | | **ESS=39.6** | | **ESS=169.1** | | **ESS=98.9** | |
|  | **Stat.** | **Stat.** | **SMD** | **Stat.** | **SMD** | **Stat.** | **SMD** | **Stat.** | **SMD** |
| Age, y, mean (SD) | 56.3 (12.0) | 60.3 (13.3) | 0.308 | 62.8 (12.1) | 0.531 | 59.4 (14.3) | 0.226 | 56.3 (12.0) | 0.000 |
| Male sex, % | 67.3 | 66.0 | 0.027 | 73.9 | 0.145 | 69.0 | 0.036 | 67.3 | 0.000 |
| IPI score, %* |  |  |  |  |  |  |  |  |  |
| 0–2 | 54.5 | 58.6 | 0.162 | 38.8 | 0.318 | 50.1 | 0.161 | 54.5 | 0.000 |
| 3–4 | 45.5 | 39.8 |  | 61.2 |  | 49.0 |  | 45.5 |  |
| 5 | 0.0 | 0.8 |  | 0.0 |  | 1.0 |  | 0.0 |  |
| Missing | 0.0 | 0.8 |  | 0.0 |  | 0.0 |  | 0.0 |  |
| ECOG PS at screening, % |  |  |  |  |  |  |  |  |  |
| 0 | 41.6 | 40.6 | 0.178 | 42.2 | 0.012 | 35.1 | 0.133 | 41.6 | 0.000 |
| 1 | 58.4 | 57.8 |  | 57.8 |  | 64.9 |  | 58.4 |  |
| 2 | 0.0 | 1.6 |  | 0.0 |  | 0.0 |  | 0.0 |  |
| Disease stage, % |  |  |  |  |  |  |  |  |  |
| I or II | 14.9 | 27.0 | 0.304 | 18.1 | 0.087 | 18.6 | 0.099 | 14.9 | 0.000 |
| III or IV | 85.1 | 72.3 |  | 81.9 |  | 81.4 |  | 85.1 |  |
| Missing | 0.0 | 0.8 |  | 0.0 |  | 0.0 |  | 0.0 |  |
| Tumor burden based on SPD before LDC, cm^2^, mean (SD)^†^ | 50.4 (43.7) | 43.7 (48.1) | 0.142 | 50.4 (44.1) | 0.000 | 50.4 (43.8) | 0.000 | 50.4 (43.9) | 0.000 |
|  |  |  |  |  |  |  |  |  |  |
| Secondary CNS disease at time of treatment, % |  |  |  |  |  |  |  |  |  |
| No | 100.0 | 97.7 | 0.219 | 100.0 | 0.000 | 100.0 | 0.000 | 100.0 | 0.000 |
| Yes | 0.0 | 2.3 |  | 0.0 |  | 0.0 |  | 0.0 |  |
| Extranodal disease, % |  |  |  |  |  |  |  |  |  |
| No | 30.7 | 46.9 | 0.344 | 30.7 | 0.000 | 30.7 | 0.000 | 30.7 | 0.000 |
| Yes | 69.3 | 52.3 |  | 69.3 |  | 69.3 |  | 69.3 |  |
| Missing | 0.0 | 0.8 |  | 0.0 |  | 0.0 |  | 0.0 |  |
| Bulky disease, % |  |  |  |  |  |  |  |  |  |
| No | 83.2 | 87.9 | 0.155 | 83.2 | 0.000 | 83.2 | 0.000 | 83.2 | 0.000 |
| Yes | 16.8 | 11.3 |  | 16.8 |  | 16.8 |  | 16.8 |  |
| Missing | 0.0 | 0.8 |  | 0.0 |  | 0.0 |  | 0.0 |  |
| Disease histology, % |  |  |  |  |  |  |  |  |  |
| DLBCL^‡^ | 76.2 | 71.1 | 0.242 | 76.3 | 0.000 | 76.3 | 0.000 | 76.3 | 0.000 |
| DLBCL tFL | 15.8 | 22.3 |  | 15.8 |  | 15.8 |  | 15.8 |  |
| PMBCL | 7.9 | 5.5 |  | 7.9 |  | 7.9 |  | 7.9 |  |
| FL3B | 0.0 | 1.2 |  | 0.0 |  | 0.0 |  | 0.0 |  |
| No. of lines of prior therapy, %^§^ |  |  |  |  |  |  |  |  |  |
| 1 | 3.0 | 0.8 | 0.179 | 1.2 | 0.347 | 0.6 | 0.191 | 3.0 | 0.000 |
| 2 | 27.7 | 25.0 |  | 43.5 |  | 30.5 |  | 27.7 |  |
| ≥3 | 69.3 | 73.8 |  | 55.3 |  | 68.9 |  | 69.3 |  |
| Missing | 0.0 | 0.4 |  | 0.0 |  | 0.0 |  | 0.0 |  |
| Prior allo-HSCT, % | 0.0 | 2.7 | 0.237 | 0.0 | 0.000 | 0.0 | 0.000 | 0.0 | 0.000 |
| Prior auto-HSCT, % | 24.8 | 33.2 | 0.186 | 24.8 | 0.000 | 24.8 | 0.000 | 24.8 | 0.000 |
| Bridging therapy, % |  |  |  |  |  |  |  |  |  |
| No | 100.0 | 41.4 | 1.682 | 100.0 | 0.000 | 33.7 | 1.986 | 35.9 | 1.889 |
| Yes | 0.0 | 58.6 |  | 0.0 |  | 66.3 |  | 64.1 |  |
| R/R to last therapy, %^¶^ |  |  |  |  |  |  |  |  |  |
| Relapsed | 20.8 | 35.9 | 0.359 | 20.8 | 0.000 | 20.8 | 0.000 | 20.8 | 0.000 |
| Refractory | 79.2 | 61.7 |  | 79.3 |  | 79.2 |  | 79.2 |  |
| Missing | 0.0 | 2.3 |  | 0.0 |  | 0.0 |  | 0.0 |  |
| CrCl before LDC, %^#^ |  |  |  |  |  |  |  |  |  |
| <60 mL/min | 0.0 | 19.1 | 0.688 | 16.4 | 0.626 | 18.0 | 0.662 | 0.0 | 0.000 |
| ≥60 mL/min | 100.0 | 80.9 |  | 83.6 |  | 82.0 |  | 100.0 |  |
| LVEF at screening, %^#^ |  |  |  |  |  |  |  |  |  |
| <50% | 0.0 | 5.1 | 0.327 | 5.3 | 0.333 | 4.0 | 0.288 | 0.0 | 0.000 |
| ≥50% | 100.0 | 94.9 |  | 94.7 |  | 96.0 |  | 100.0 |  |
| Pre-leukapheresis ALC (10^9^/L), %^#^ |  |  |  |  |  |  |  |  |  |
| <0.1 | 0.0 | 0.4 | 0.091 | 0.0 | 0.000 | 0.1 | 0.054 | 0.0 | 0.000 |
| ≥0.1 | 100.0 | 94.1 |  | 95.0 |  | 94.2 |  | 100.0 |  |
| Missing | 0.0 | 5.5 |  | 5.0 |  | 5.7 |  | 0.0 |  |
| Statistics, % |  |  |  |  |  |  |  |  |  |
| Factors with SMD <0.2 | NA | 44.4 | NA | 72.2 | NA | 77.8 | NA | 94.4 | NA |
| Factors with SMD <0.1 | NA | 11.1 | NA | 66.7 | NA | 61.1 | NA | 94.4 | NA |

ALC, absolute lymphocyte count; allo-HSCT, allogeneic hematopoietic stem cell transplantation; auto-HSCT, autologous hematopoietic stem cell transplantation; axi-cel, axicabtagene ciloleucel; CNS, central nervous system; CRR, complete response rate; CrCl, creatinine clearance; DLBCL, diffuse large B-cell lymphoma; ECOG PS, Eastern Cooperative Oncology Group performance status; ESS, effective sample size; FL3B, follicular lymphoma grade 3B; HGBCL, high-grade B-cell lymphoma; IPI, International Prognostic Index; LBCL, large B-cell lymphoma; liso-cel, lisocabtagene maraleucel; LDC, lymphodepleting chemotherapy; LVEF, left ventricular ejection fraction; MAIC, matching-adjusted indirect comparison; mITT, modified intention to treat; N, sample size; NA, not applicable; NOS, not otherwise specified; PMBCL, primary mediastinal B-cell lymphoma; R/R, relapsed or refractory; SA1, sensitivity analysis 1; SA2, sensitivity analysis 2; SD, standard deviation; SMD, standard mean difference; SPD, sum of the product of perpendicular diameters; Stat., statistic; tFL, transformed from follicular lymphoma; tiNHL, transformed indolent non-Hodgkin lymphoma.

*Per ZUMA-1 categorization.

^†^Per investigator assessment.

^‡^Includes DLBCL NOS, HGBCL, and tiNHL for TRANSCEND; includes DLBCL NOS and HGBCL for ZUMA-1.

^§^Per ZUMA-1, salvage chemotherapy and auto-HSCT were considered separate regimens.

^¶^Per ZUMA-1, refractory was defined as best response to last therapy of progressive disease or stable disease and relapsed defined as best response to last therapy of partial response or complete response.

^#^Per ZUMA-1 eligibility criteria, all patients enrolled in ZUMA-1 had CrCl ≥60 mL/min before LDC, LVEF ≥50% at screening, and pre-leukapheresis ALC ≥0.1×10^9^/L.

Supplemental Table 4. Comparison of clinical factors and SMDs before and after MAIC for ORR

| **Clinical factor** | **ORR** | | | | | | | | |
| --- | --- | --- | --- | --- | --- | --- | --- | --- | --- |
|  | **ZUMA-1  (axi-cel) phase 2 mITT set** | **TRANSCEND (liso-cel) LBCL efficacy set** | | | | | | | |
|  |  | **Before MAIC  (naïve)** | **After MAIC (initial)** | | | **After MAIC  (SA1)** | | **After MAIC  (SA2)** | |
| **N/ESS** | **N=101** | **N=256** | **ESS=42.1** | | | **ESS=150.3** | | **ESS=98.9** | |
|  | **Stat.** | **Stat.** | **SMD** | **Stat.** | **SMD** | **Stat.** | **SMD** | **Stat.** | **SMD** |
| Age, y, mean (SD) | 56.3 (12.0) | 60.3 (13.3) | 0.308 | 60.4 (12.4) | 0.331 | 58.1 (13.8) | 0.137 | 56.3 (12.0) | 0.000 |
| Male sex, % | 67.3 | 66.0 | 0.027 | 80.2 | 0.295 | 74.2 | 0.152 | 67.3 | 0.000 |
| IPI score, %* |  |  |  |  |  |  |  |  |  |
| 0–2 | 54.5 | 58.6 | 0.162 | 46.9 | 0.152 | 53.7 | 0.137 | 54.5 | 0.000 |
| 3–4 | 45.5 | 39.8 |  | 53.1 |  | 45.3 |  | 45.5 |  |
| 5 | 0.0 | 0.8 |  | 0.0 |  | 0.9 |  | 0.0 |  |
| Missing | 0.0 | 0.8 |  | 0.0 |  | 0.0 |  | 0.0 |  |
| ECOG PS at screening, % |  |  |  |  |  |  |  |  |  |
| 0 | 41.6 | 40.6 | 0.178 | 47.2 | 0.113 | 37.7 | 0.08 | 41.6 | 0.000 |
| 1 | 58.4 | 57.8 |  | 52.8 |  | 62.3 |  | 58.4 |  |
| 2 | 0.0 | 1.6 |  | 0.0 |  | 0.0 |  | 0.0 |  |
| Disease stage, % |  |  |  |  |  |  |  |  |  |
| I or II | 14.9 | 27.0 | 0.304 | 14.9 | 0.000 | 14.9 | 0.000 | 14.9 | 0.000 |
| III or IV | 85.1 | 72.3 |  | 85.1 |  | 85.1 |  | 85.1 |  |
| Missing | 0.0 | 0.8 |  | 0.0 |  | 0.0 |  | 0.0 |  |
| Tumor burden based on SPD before LDC, cm^2^, mean (SD)^†^ | 50.4 (43.7) | 43.7 (48.1) | 0.142 | 50.4 (44.1) | 0.000 | 50.4 (43.8) | 0.000 | 50.4 (43.9) | 0.000 |
| Secondary CNS disease at time of treatment, % |  |  |  |  |  |  |  |  |  |
| No | 100.0 | 97.7 | 0.219 | 100.0 | 0.000 | 100.0 | 0.000 | 100.0 | 0.000 |
| Yes | 0.0 | 2.3 |  | 0.0 |  | 0.0 |  | 0.0 |  |
| Extranodal disease, % |  |  |  |  |  |  |  |  |  |
| No | 30.7 | 46.9 | 0.344 | 38.7 | 0.169 | 38.0 | 0.155 | 30.7 | 0.000 |
| Yes | 69.3 | 52.3 |  | 61.3 |  | 62.0 |  | 69.3 |  |
| Missing | 0.0 | 0.8 |  | 0.0 |  | 0.0 |  | 0.0 |  |
| Bulky disease, % |  |  |  |  |  |  |  |  |  |
| No | 83.2 | 87.9 | 0.155 | 88.9 | 0.165 | 85.3 | 0.057 | 83.2 | 0.000 |
| Yes | 16.8 | 11.3 |  | 11.1 |  | 14.7 |  | 16.8 |  |
| Missing | 0.0 | 0.8 |  | 0.0 |  | 0.0 |  | 0.0 |  |
| Disease histology, % |  |  |  |  |  |  |  |  |  |
| DLBCL^‡^ | 76.2 | 71.1 | 0.242 | 59.3 | 0.372 | 67.8 | 0.198 | 76.3 | 0.000 |
| DLBCL tFL | 15.8 | 22.3 |  | 28.1 |  | 23.0 |  | 15.8 |  |
| PMBCL | 7.9 | 5.5 |  | 12.6 |  | 9.2 |  | 7.9 |  |
| FL3B | 0.0 | 1.2 |  | 0.0 |  | 0.0 |  | 0.0 |  |
| No. of lines of prior therapy, %^§^ |  |  |  |  |  |  |  |  |  |
| 1 | 3.0 | 0.8 | 0.179 | 1.5 | 0.370 | 0.8 | 0.171 | 3.0 | 0.000 |
| 2 | 27.7 | 25.0 |  | 45.0 |  | 30.3 |  | 27.7 |  |
| ≥3 | 69.3 | 73.8 |  | 53.5 |  | 68.9 |  | 69.3 |  |
| Missing | 0.0 | 0.4 |  | 0.0 |  | 0.0 |  | 0.0 |  |
| Prior allo-HSCT, % | 0.0 | 2.7 | 0.237 | 0.0 | 0.000 | 0.0 | 0.000 | 0.0 | 0.000 |
| Prior auto-HSCT, % | 24.8 | 33.2 | 0.186 | 24.8 | 0.000 | 24.8 | 0.000 | 24.8 | 0.000 |
| Bridging therapy, % |  |  |  |  |  |  |  |  |  |
| No | 100.0 | 41.4 | 1.682 | 100.0 | 0.000 | 34.7 | 1.941 | 35.9 | 1.889 |
| Yes | 0.0 | 58.6 |  | 0.0 |  | 65.3 |  | 64.1 |  |
| R/R to last therapy, %^¶^ |  |  |  |  |  |  |  |  |  |
| Relapsed | 20.8 | 35.9 | 0.359 | 20.8 | 0.000 | 20.8 | 0.000 | 20.8 | 0.000 |
| Refractory | 79.2 | 61.7 |  | 79.2 |  | 79.2 |  | 79.2 |  |
| Missing | 0.0 | 2.3 |  | 0.0 |  | 0.0 |  | 0.0 |  |
| CrCl before LDC, %^#^ |  |  |  |  |  |  |  |  |  |
| <60 mL/min | 0.0 | 19.1 | 0.688 | 0.0 | 0.000 | 0.0 | 0.000 | 0.0 | 0.000 |
| $\geq$60 mL/min | 100.0 | 80.9 |  | 100.0 |  | 100.0 |  | 100.0 |  |
| LVEF at screening, %^#^ |  |  |  |  |  |  |  |  |  |
| <50% | 0.0 | 5.1 | 0.327 | 9.8 | 0.467 | 5.4 | 0.338 | 0.0 | 0.000 |
| ≥50% | 100.0 | 94.9 |  | 90.2 |  | 94.6 |  | 100.0 |  |
| Pre-leukapheresis ALC (10^9^/L), %^#^ |  |  |  |  |  |  |  |  |  |
| <0.1 | 0.0 | 0.4 | 0.091 | 0.0 | 0.000 | 0.2 | 0.070 | 0.0 | 0.000 |
| ≥0.1 | 100.0 | 94.1 |  | 92.4 |  | 92.2 |  | 100.0 |  |
| Missing | 0.0 | 5.5 |  | 7.6 |  | 7.5 |  | 0.0 |  |
|  |  |  |  |  |  |  |  |  |  |
| Statistics, % |  |  |  |  |  |  |  |  |  |
| Factors with SMD <0.2 | NA | 44.4 | NA | 72.2 | NA | 88.9 | NA | 94.4 | NA |
| Factors with SMD <0.1 | NA | 11.1 | NA | 50.0 | NA | 55.6 | NA | 94.4 | NA |

ALC, absolute lymphocyte count; allo-HSCT, allogeneic hematopoietic stem cell transplantation; auto-HSCT, autologous hematopoietic stem cell transplantation; axi-cel, axicabtagene ciloleucel; CNS, central nervous system; CrCl, creatinine clearance; DLBCL, diffuse large B-cell lymphoma; ECOG PS, Eastern Cooperative Oncology Group performance status; ESS, effective sample size; FL3B, follicular lymphoma grade 3B; HGBCL, high-grade B-cell lymphoma; IPI, International Prognostic Index; LBCL, large B-cell lymphoma; liso-cel, lisocabtagene maraleucel; LDC, lymphodepleting chemotherapy; LVEF, left ventricular ejection fraction; MAIC, matching-adjusted indirect comparison; mITT, modified intention to treat; N, sample size; NA, not applicable; NOS, not otherwise specified; ORR, objective response rate; PMBCL, primary mediastinal B-cell lymphoma; R/R, relapsed or refractory; SA1, sensitivity analysis 1; SA2, sensitivity analysis 2; SD, standard deviation; SMD, standard mean difference; SPD, sum of the product of perpendicular diameters; Stat., statistic; tFL, transformed follicular lymphoma; tiNHL, transformed indolent non-Hodgkin lymphoma.

*Per ZUMA-1 categorization.

^†^Per investigator assessment.

^‡^Includes DLBCL NOS, HGBCL, and tiNHL for TRANSCEND; includes DLBCL NOS and HGBCL for ZUMA-1.

^§^Per ZUMA-1, salvage chemotherapy and auto-HSCT were considered separate regimens.

^¶^Per ZUMA-1, refractory was defined as best response to last therapy of progressive disease or stable disease and relapsed defined as best response to last therapy of partial response or complete response.

^#^Per ZUMA-1 eligibility criteria, all patients enrolled in ZUMA-1 had CrCl ≥60 mL/min before LDC, LVEF ≥50% at screening, and pre-leukapheresis ALC ≥0.1×10^9^/L.

Supplemental Table 5. Comparison of clinical factors and SMDs before and after MAIC for safety analysis

| Clinical factor | Safety analysis | | | | | | |
| --- | --- | --- | --- | --- | --- | --- | --- |
|  | ZUMA-1  (axi-cel) phase 1/2 safety analysis set | TRANSCEND (liso-cel) LBCL-treated set | | | | | |
|  |  | Before MAIC  **(naive)** | | After MAIC  **(initial)** | | After MAIC  **(sensitivity)** | |
| N/ESS | N=108 | N=269 | | ESS=63.0 | | ESS=209.9 | |
|  | Stat. | Stat. | SMD | Stat. | SMD | Stat. | SMD |
| Age, y, mean (SD) | 56.0 (12.5) | 60.1 (13.3) | 0.312 | 62.5 (11.3) | 0.540 | 60.1 (13.6) | 0.305 |
| ECOG PS at screening, % |  |  |  |  |  |  |  |
| 0 | 42.6 | 40.9 | 0.175 | 46.0 | 0.069 | 41.9 | 0.015 |
| 1 | 57.4 | 57.6 |  | 54.0 |  | 58.1 |  |
| 2 | 0.0 | 1.5 |  | 0.0 |  | 0.0 |  |
| Tumor burden based on SPD before LDC, cm^2^, mean (SD)* | 48.9 (42.8) | 45.2 (56.1) | 0.069 | 48.8 (43.0) | 0.000 | 48.9 (42.9) | 0.000 |
| Secondary CNS disease at time of treatment, % |  |  |  |  |  |  |  |
| No | 100.0 | 97.4 | 0.231 | 100.0 | 0.000 | 100 | 0.000 |
| Yes | 0.0 | 2.6 |  | 0.0 |  | 0.0 |  |
| No. of lines of prior therapy, %^†^ |  |  |  |  |  |  |  |
| 1 | 2.8 | 0.7 | 0.164 | 2.7 | 0.000 | 2.7 | 0.000 |
| 2 | 26.9 | 24.5 |  | 26.9 |  | 26.9 |  |
| ≥3 | 70.4 | 74.3 |  | 70.4 |  | 70.4 |  |
| Missing | 0.0 | 0.4 |  | 0.0 |  | 0.0 |  |
| Prior allo-HSCT, % | 0.0 | 3.3 | 0.263 | 0.0 | 0.000 | 0.0 | 0.000 |
| Prior auto-HSCT, % | 26.9 | 33.5 | 0.143 | 39.1 | 0.262 | 29.3 | 0.053 |
| Bridging therapy, % |  |  |  |  |  |  |  |
| No | 100.0 | 40.9 | 1.7 | 100.0 | 0.000 | 41.0 | 1.696 |
| Yes | 0.0 | 59.1 | 1.7 | 0.0 |  | 59.0 |  |
| Baseline^‡^ cytopenia, % |  |  |  |  |  |  |  |
| Grade ≥3 anemia | 2.8 | 5.9 | 0.154 | 2.8 | 0.000 | 2.8 | 0.000 |
| Grade ≥3 neutropenia | 3.7 | 7.1 | 0.149 | 3.7 | 0.000 | 3.7 | 0.000 |
| Grade ≥3 thrombocytopenia | 3.7 | 6.7 | 0.135 | 3.7 | 0.000 | 3.7 | 0.000 |
| Statistics, % |  |  |  |  |  |  |  |
| Factors with SMD <0.2 | NA | 63.6 | NA | 81.8 | NA | NA | 81.8 |
| Factors with SMD <0.1 | NA | 9.1 | NA | 81.8 | NA | NA | 81.8 |

Allo-HSCT, allogeneic hematopoietic stem cell transplantation; auto-HSCT, autologous hematopoietic stem cell transplantation; axi-cel, axicabtagene ciloleucel; CNS, central nervous system; ECOG PS, Eastern Cooperative Oncology Group performance status; ESS, effective sample size; LBCL, large B-cell lymphoma; liso-cel, lisocabtagene maraleucel; LDC, lymphodepleting chemotherapy; MAIC, matching-adjusted indirect comparison; N, sample size; NA, not applicable; SD, standard deviation; SMD, standard mean difference; SPD, sum of the product of perpendicular diameters; Stat., statistic.

*Per investigator assessment.

^†^Per ZUMA-1 categorization.

^‡^Baseline anemia, neutropenia, and thrombocytopenia were assessed by laboratory values according to Common Terminology Criteria for Adverse Events, v4.03. Data for TRANSCEND were from screening and those for ZUMA-1 were from before LDC.
